# Substrate channeling in oxylipin biosynthesis through a protein complex in the plastid envelope of *Arabidopsis thaliana*

**DOI:** 10.1093/jxb/erz015

**Published:** 2019-01-23

**Authors:** Stephan Pollmann, Armin Springer, Sachin Rustgi, Diter von Wettstein, ChulHee Kang, Christiane Reinbothe, Steffen Reinbothe

**Affiliations:** 1 Centro de Biotecnología y Genómica de Plantas, Universidad Politécnica de Madrid (UPM) – Instituto Nacional de Investigación y Tecnología Agraria y Alimentación (INIA), Campus de Montegancedo, Pozuelo de Alarcón (Madrid), Spain; 2 Medizinische Biologie und Elektronenmikroskopisches Zentrum (EMZ), Universitätsmedizin Rostock, Rostock, Germany; 3 Department of Plant and Environmental Sciences, Pee Dee Research and Education Center, Clemson University, Florence, SC, USA; 4 Department of Crop and Soil Sciences, Washington State University, Pullman, WA, USA; 5 Molecular Plant Sciences Program, Washington State University, Pullman, WA, USA; 6 Center for Reproductive Biology, Washington State University, Pullman, WA, USA; 7 Department of Chemistry, Washington State University, Pullman, WA, USA; 8 School of Molecular Biosciences, Washington State University, Pullman, WA, USA; 9 Biomolecular Crystallography Center, Washington State University, Pullman, WA, USA; 10 Laboratoire de Génétique Moléculaire des Plantes, Université Grenoble Alpes, CEDEX, France

**Keywords:** Allene oxide synthase, allene oxide cyclase, chloroplast envelope protein complex, hydroperoxide lyase, lipoxygenase, metabolite channeling, plant defense

## Abstract

Oxygenated membrane fatty acid derivatives termed oxylipins play important roles in plant defense against biotic and abiotic cues. Plants challenged by insect pests, for example, synthesize a blend of different defense compounds that include volatile aldehydes and jasmonic acid (JA), among others. Because all oxylipins are derived from the same pathway, we investigated how their synthesis might be regulated, focusing on two closely related atypical cytochrome P450 enzymes designated CYP74A and CYP74B, respectively, allene oxide synthase (AOS) and hydroperoxide lyase (HPL). These enzymes compete for the same substrate but give rise to different products: the final product of the AOS branch of the oxylipin pathway is JA, while those of the HPL branch comprise volatile aldehydes and alcohols. AOS and HPL are plastid envelope enzymes in *Arabidopsis thaliana* but accumulate at different locations. Biochemical experiments identified AOS as a constituent of complexes also containing lipoxygenase 2 (LOX2) and allene oxide cyclase (AOC), which catalyze consecutive steps in JA precursor biosynthesis, while excluding the concurrent HPL reaction. Based on published X-ray data, the structure of this complex was modelled and amino acids involved in catalysis and subunit interactions predicted. Genetic studies identified the microRNA 319-regulated clade of TCP (TEOSINTE BRANCHED/CYCLOIDEA/PCF) transcription factor genes and CORONATINE INSENSITIVE 1 (COI1) as controlling JA production through the LOX2-AOS-AOC2 complex. Together, our results define a molecular branch point in oxylipin biosynthesis that allows fine-tuning of the plant’s defense machinery in response to biotic and abiotic stimuli.

## Teaser

Together with the interaction of miR319-regulated TCP transcription factors and the COI1 receptor, the formation of LOX2-AOS-AOC2 complexes controls key steps in jasmonic acid precursor biosynthesis, facilitating fine-tuning of the plant’s defense machinery.

## Introduction

**Fig. 1.**
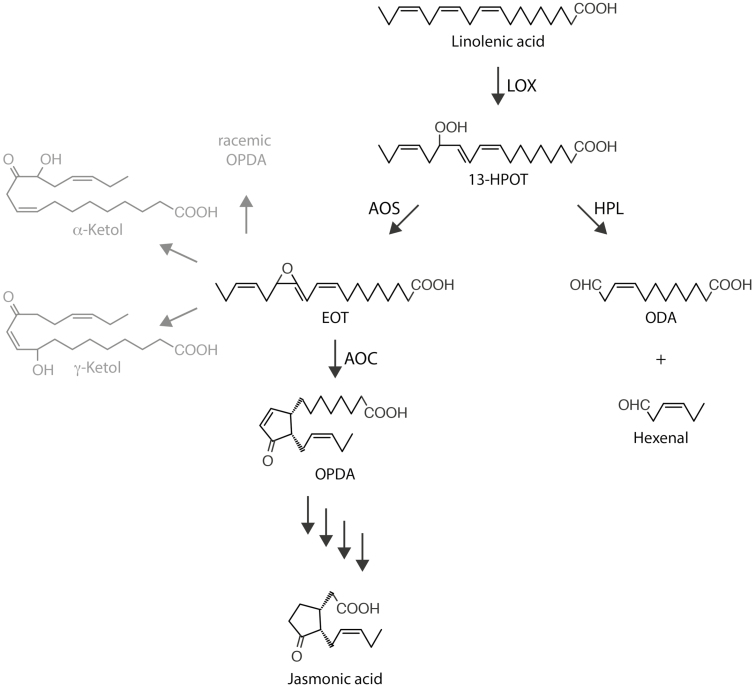
The Vick and Zimmerman pathway leading to the synthesis of jasmonic acid, and the concurrent AOS and HPL reactions. Pathway intermediates are indicated as follows: 13-HPOT, (9*Z*11*E*15*Z*13*S*)-13-hydroperoxy-9,11,15-octadecatrienoic acid; EOT, 12,13(*S*)-epoxy-9(*Z*),11,15(*Z*)-octadecatrienoic acid; ODA, 12-oxo-*cis*-9-dodecenoic acid; OPDA, *cis*-(+)-12-oxophytodienoic acid. Enzymes are indicated as follows: AOC, allene oxide cyclase; AOS, allene oxide synthase; HPL, hydroperoxy lyase; LOX, 13-lipoxygenase. Note that EOT is short-lived and spontaneously disintegrates into volatile α-ketols and γ-ketols as well as racemic OPDA.

Jasmonic acid (JA) and its derivatives are cyclopentanone compounds of widespread occurrence and ubiquitous function in plants (Böttcher and Pollmann, 2009; Reinbothe *et al.*, 2009; Wasternack and Hause, 2013; Yan *et al.*, 2013). JA biosynthesis involves the release of linolenic acid from membrane lipids by phospholipases and galactolipases, as well as 13-lipoxygenase (LOX), allene oxide synthase (AOS), and allene oxide cyclase (AOC) carrying out consecutive reactions in chloroplasts (Fig. 1). 13-LOX (EC 1.13.11.12) catalyzes the regio- and stereospecific hydroperoxidation of the C-13 atom of α-linolenic acid (α-LeA), giving rise to (13*S*)-hydroperoxylinolenic acid (13-HPOT), and AOS (EC 4.2.1.92) converts 13-HPOT to 12,13-epoxylinolenic acid (EOT). Because EOT is short-lived and spontaneously disintegrates into volatile α- and γ-ketols as well as racemic 12-oxo-phytodienoic acid (OPDA), plants make use of AOC (EC 5.3.99.6) to assure *cis*-(+)-12-oxo-phytodienoic acid (*cis*-(+)-12-OPDA) synthesis. *cis*-(+)-12-OPDA is then exported from chloroplasts to the cytosol and transported further into peroxisomes, where the final reduction and β-oxidation steps of JA biosynthesis take place. In *Arabidopsis thaliana*, six genes encode LOX isoforms, one gene encodes AOS, and four genes encode AOC enzymes. Studies in mutants have provided valuable insights into the roles of the different LOX, AOS, and AOC isoforms *in planta* (Schaller *et al.*, 2008; Schaller and Stintzi, 2009; Wasternack and Hause, 2013; Yan *et al.*, 2013).

(+)-*7-iso*-JA-Ile (JA-Ile) is the physiologically active compound of JA signaling (Fonseca *et al.*, 2009). It triggers changes in gene expression including the activation of defense genes and inhibition of photosynthetic genes (Reinbothe *et al.*, 1993a, b; Rustgi *et al.*, 2014). Key regulatory elements in JA signaling involve the F-box protein COI1 (CORONATINE INSENSITIVE 1) acting as the JA-Ile receptor (Xie *et al.*, 1998; Yan *et al.*, 2009), the E3 ubiquitin-ligase Skp-Cullin-F-box complex SCF^COI1^, and the JASMONATE ZIM-domain (JAZ) transcriptional repressors, which normally suppress the expression of JA response genes (Chini *et al.*, 2007; Thines *et al.*, 2007; Chung *et al.*, 2008). Binding of JA-Ile to COI1 elicits the degradation of JAZ transcriptional repressors through the 26S proteasome and permits the expression of JA response genes, driven by a number of MYC transcription factors (Chini *et al.*, 2009; Fernández-Calvo *et al.*, 2011; Hoffmann *et al.*, 2011; Schweizer *et al.*, 2013).

The Vick and Zimmerman pathway through which JA is produced has a number of branch points (Fig. 1). The question of how the flow of metabolites through the different branches is regulated in time and space is largely unanswered. In particular, several reactions compete for 13-HPOT *in planta* (Wasternack and Hause, 2013; Griffiths, 2015; Nilsson *et al.*, 2016). One such reaction is catalyzed by fatty acid hydroperoxide lyase (HPL), which cleaves 13-HPOT into *Z*-3-hexenal and 12-oxo-*cis*-9-dodecenoic acid (ODA), of which *cis*-3-hexenal and the corresponding alcohol are volatile compounds with a role in herbivore deterrence (Bate and Rothstein, 1998; Blée, 1998; Croft *et al.*, 1993; Wu and Baldwin, 2010) (Fig. 1).

Here, we report on the identification of a protein complex comprising LOX2, AOS, and AOC2 in the plastid envelope of Arabidopsis chloroplasts that channels α-LeA into JA biosynthesis. Because the expression of LOX2, AOS, and AOC2 is under the control of JA-responsive microRNAs and *COI1*, a mechanism is suggested that boosts JA production over aldehyde production for rapid local and systemic activation of defense genes in plants.

## Materials and methods

### Plant growth

Wild-type *A. thaliana* (Col-0) and *jaw-*D (Palatnik *et al.*, 2003; Schommer *et al.*, 2008), *aos* (Park *et al.*, 2002; von Malek *et al.*, 2002), *coi1* (Feys *et al.*, 1994), *lox2* (Seltmann *et al.*, 2010), *aoc2* (Stenzel *et al.*, 2012), and *jar1* (Staswick *et al.*, 1992) genotypes were used in this study. Homozygous *aos* plants were obtained by hand-pollinating stigmas of *aos* flowers with *aos* plants that had been generated by spraying flowering homozygous plants with 45 µM methyl jasmonate (MeJA). Seeds from an F_2_ population segregating for the *coi1* mutation were obtained as described by Feys *et al.* (1994). Plants were grown at 25 °C under standard conditions under either continuous white light illumination provided by fluorescent bulbs (30 W/m^2^) or 16 h light/8 h dark cycles.

### Generation of transgenic lines expressing AOS or HPL with C-terminal (His)_6_ or FLAG tags

Transgenic plants were generated expressing C-terminal hexa-histidine (His)_6_, FLAG-tagged AOS or FLAG-tagged HPL as described in Supplementary Protocol S1 at JXB online and used for affinity purification of proteins interacting with AOS or HPL *in planta*.

### Production of proteins and import into chloroplasts

cDNA clones for *AOS* (Laudert *et al.*, 1996) and *HPL* (Froehlich *et al.*, 2001) were cloned into appropriate vectors, allowing for their purification as (His)_6_- or FLAG-tagged proteins. For routine chloroplast import assays, ^35^S-methionine- or ^14^C-leucine-labeled proteins were produced by coupled *in vitro* transcription/translation in wheat germ extracts. Radiolabeled proteins were added to 50 µl import assays consisting of 25 µl of double-concentrated import buffer, 10 µl of a plastid suspension containing 5 × 10^7^ Arabidopsis chloroplasts, and 2.5 mM Mg-ATP. All import reactions were performed at 23 °C for 15 min in darkness. Post-import protease treatment of plastids with thermolysin or trypsin and extraction of membranes with sodium carbonate (pH 11) or 1 M NaCl were carried out as described (Cline *et al.*, 1984; Kessler and Blobel, 1996). Trypsin quenching was monitored by western blotting using TIC110 as a diagnostic marker and employing a trypsin inhibitor (Jackson *et al.*, 1998). Plastid subfractionation into envelopes, stroma, and thylakoids was done according to Li *et al.* (1991). Protein was extracted and precipitated with trichloroacetic acid [5% (w/v) final concentration], resolved by SDS-PAGE on 10–20% (w/v) polyacrylamide gradients (Laemmli, 1970), and detected by autoradiography. Because the presence of the tags only marginally affected the molecular masses of the tagged proteins [peptide mass of 1013 g mol^−1^ for the (His)_6_ tag and 841 g mol^−1^ for the FLAG tag], molecular masses are reported uniformly throughout this paper and refer to the untagged proteins.

### Isolation of AOS-containing and HPL-containing higher molecular mass complexes

AOS-(His)_6_ and HPL-(His)_6_ were imported into isolated Arabidopsis chloroplasts, and proteins interacting with AOS-(His)_6_- or HPL-(His)_6_ were purified from detergent-solubilized envelopes by Ni-NTA affinity chromatography (Qiagen). For comparison, AOS- or HPL-containing plastid envelope protein complexes were purified from plants expressing FLAG-tagged AOS (AOS-FLAG) or HPL (HPL-FLAG) (see Supplementary Protocol S1). Both types of complexes were subjected to size exclusion chromatography on Superose 6 (column model HR10/10, GE Healthcare) and individual fractions were harvested and traced for the presence of AOS or HPL by western blotting using AOS-, HPL-, or FLAG-specific antibodies (Laudert *et al.*, 1996; see also Supplementary Fig. S1) and an enhanced chemiluminescence kit (ECL, GE Healthcare).

### Cross-linking

Cross-linking was carried out using ^125^I-*N*-[4-(*p*-azidosalicylamido)butyl]-3′(2-pyridyldithio)propionamide (^125^I-APDP)-derivatized precursors, essentially as previously described by Ma *et al.* (1996). Final protein samples were separated by reducing or non-reducing SDS-PAGE and ^125^I-labeled proteins were detected by autoradiography.

### Enzyme activity measurements

Activity measurements using the isolated native and reconstituted complexes or free enzymes were carried out as described in Supplementary Protocol S1. HPLC and GC-MS analyses used to identify and quantify substrates and products of the LOX2, AOS, and AOC2 reactions were performed according to Holtman *et al.* (1997) and Zerbe *et al.* (2007). Capillary chiral GC analysis was used to demonstrate the optical purity of the *cis*-(+)-enantiomer (>95%), which was reconfirmed by TLC, HPLC, and GC analyses with a synthetic standard (Zerbe *et al.*, 2007).

### Yeast two-hybrid screens

Split-ubiquitin yeast two-hybrid screens for membrane-bound proteins were carried out according to the manufacturer’s instructions using a commercial system (Dualsystems Biotech AG, Switzerland). Vector construction is described in Supplementary Protocol S1.

### Molecular modelling

Molecular modeling methods and tools are described in Supplementary Protocol S1.

### Miscellaneous

**Fig. 3.**
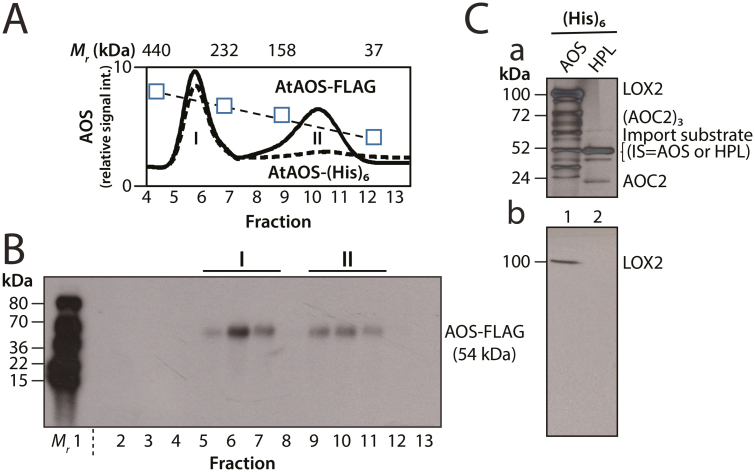
Detection of AOS complexes *in vitro* and *in planta*. (A) Gel filtration elution profile of AOS-FLAG in protein extracts of transgenic plants expressing FLAG-tagged AOS (solid line) and in isolated chloroplasts after *in vitro* import of AOS-(His)_6_ (dashed line). AOS-FLAG was quantified by western blotting using FLAG-specific antibodies and an enhanced chemiluminescence (ECL) system. Similarly, AOS-(His)_6_ signals were quantified by either western blotting using FLAG-specific antibodies and ECL detection, or radioactivity measurements in the case of ^35^S-AOS-(His)_6_. For easier comparison, the different curves were normalized and values are expressed as relative signal intensities (int.). The positions of apo-ferritin (440 kDa), catalase (232 kDa), aldolase (158 kDa), and carbonic anhydrase (37 kDa), used as molecular size standards, are indicated (squares and dotted line). (B) Western blot analysis of AOS-FLAG. Positions of purified ^14^Leu-labeled tomato heat shock proteins used as molecular size markers are indicated (*M*_*r*_*).* (C) Panel a, SDS-PAGE pattern of plastid envelope proteins co-purifying with AOS-(His)_6_ (lane 1) and HPL-(His)_6_ (lane 2). The indicated bands were identified by protein sequencing. Panel b, western blotting to identify LOX2 among the proteins detected in panel a.

Western blotting was carried out according to Towbin *et al.* (1979), using the indicated antisera and enhanced chemiluminescence (ECL, GE Healthcare) or anti-rabbit, anti-goat alkaline phosphatase systems. Molecular mass standards used in Fig. 3B were ^14^C-Leu-labeled heat shock proteins from tomato (Nover and Scharf, 1984).

## Results

### *In vitro* import into chloroplasts and membrane targeting of AOS and HPL

**Fig. 2.**
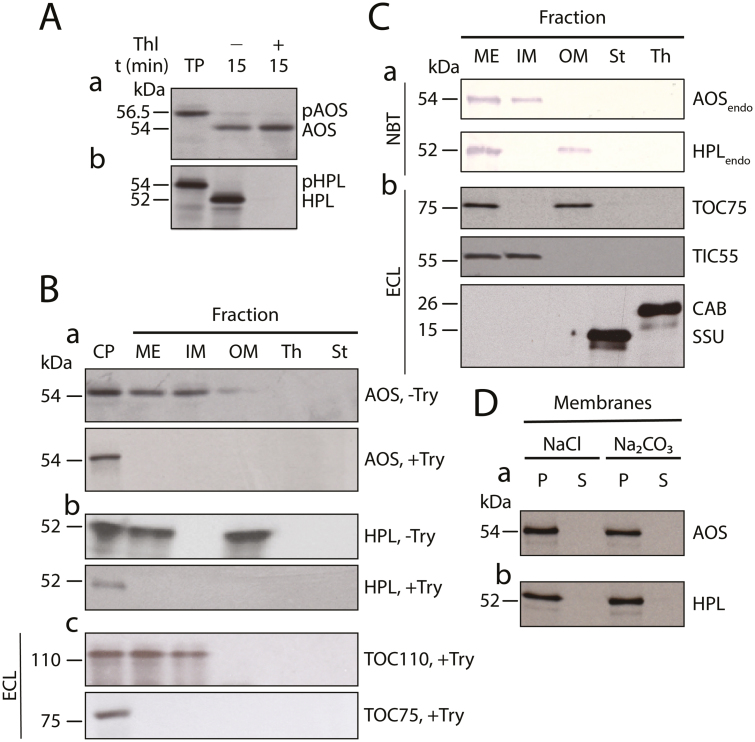
*In vitro* import and differential membrane binding of AOS and HPL in chloroplasts. (A) Levels of ^35^S-Met-labelled ^35^S-AOS (a) and ^35^S-HPL (b) before and after import into isolated Arabidopsis chloroplasts. Thl, thermolysin; TP, translation product. The positions of precursor proteins (pAOS and pHPL) and mature proteins (AOS, HPL) are indicated. (B) Detection by SDS-PAGE and autoradiography of ^35^S-AOS (a) and ^35^S-HPL (b) in trypsin (Try)-treated (+Try) and untreated (-Try) mixed outer and inner plastid envelopes (ME), inner plastid envelope (IM), outer plastid envelope (OM), thylakoids (Th), and stroma (St). CP, Chloroplast reference fraction prior to import and protease treatment. The western blot in panel c shows the levels of the inner chloroplast envelope translocon protein TIC110 and the outer chloroplast envelope protein TOC75 in non-trypsin-treated chloroplasts (CP) versus trypsin-treated chloroplasts containing imported ^35^S-AOS/HPL and respective subfractions. Signal detection was made with an enhanced chemiluminescence (ECL) system. (C) Western blot analysis of the endogenous AOS (AOS_endo_) and HPL (HPL_endo_) (predicted molecular masses 54.5 kDa and 51.3 kDa, respectively; panel a) compared with TOC75, the translocon at the inner chloroplast envelope membrane protein TIC55, the chlorophyll *a/b* binding protein LHCII (CAB), and the small subunit of ribulose-1,5-bisphosphate carboxylase/oxygenase (SSU) (panel b) in the indicated fractions of non-trypsin-treated chloroplasts. Signal detection was made with either ECL or alkaline phosphatase-5-bromo-4-chloro-3-indolyl phosphate/nitro blue tetrazolium (NBT)-based systems, as indicated. (D) Membrane binding of imported ^35^S-AOS (a) and ^35^S-HPL (b), as assessed by their extractability by 1 M NaCl and 0.1 M Na_2_CO_3_, pH 11. Both pellet (P) and supernatant (S) fractions, obtained after sedimentation of the membranes, were tested by SDS-PAGE and autoradiography for the two labeled proteins.

AOS and HPL from *A. thaliana* and other plant species belong to the family of atypical cytochrome P450s, designated CYP74. AOS (CYP74A) and HPL (CYP74B) require neither oxygen nor NADPH-dependent cytochrome P450 reductase for activity and thus are non-canonical P450s (Song and Brash, 1991; Laudert *et al.*, 1996; Froehlich *et al.*, 2001). Previously described cDNAs for AtAOS and AtHPL (Laudert *et al.*, 1996; Froehlich *et al.*, 2001) were used for coupled *in vitro* transcription/translation in wheat germ extracts. The ^35^S-methionine- or ^14^C-leucine-labeled proteins were then added to Arabidopsis chloroplasts that had been isolated by Percoll/sucrose density gradient centrifugation. Import experiments revealed that both ^35^S-AOS and ^35^S-HPL were taken up by isolated chloroplasts and processed by cleavage of their transit peptides (Fig. 2A). The resulting mature enzymes were targeted to different locations (Fig. 2B–D): whereas ^35^S-AOS was targeted to the inner envelope of chloroplasts, ^35^S-HPL was localized in the outer envelope fraction (Fig. 2A, B). Western blotting using monospecific antibodies to AOS and HPL (Supplementary Fig. S1) as well as GFP tagging confirmed the differential localization of the endogenous AOS and HPL *in planta* (Fig. 2C; Supplementary Fig. S2). The *in vitro*-imported ^35^S-AOS and ^35^S-HPL were degraded by added trypsin, but only ^35^S-HPL was sensitive to thermolysin (Fig. 2A, B). Thermolysin is a protease that degrades surface-exposed proteins of the outer plastid envelope, whereas trypsin is known to access the intermembrane space between the outer and inner envelope (Cline *et al.*, 1984; Kessler and Blobel, 1996). Thus, AOS is likely to face the intermembrane space, as it was not degraded by thermolysin, whereas HPL is exposed to the cytosolic side of the outer plastid envelope and was, thus, sensitive to thermolysin. Western blot experiments revealed the co-localization of both the imported ^35^S-labeled AOS and endogenous AOS with two translocon proteins of the inner chloroplast envelope, TIC55 and TIC110, and that of the imported ^35^S-labeled HPL and endogenous HPL with the translocon protein of the outer chloroplast envelope, TOC75 (Fig. 2C). Using TIC110 as a diagnostic marker, any post-import degradation of ^35^S-AOS or ^35^S-HPL could be excluded (Fig. 2B). TIC110 is a very sensitive marker for monitoring the trypsin digestion procedure and is easily degraded if no precautions are taken, such as the inclusion of a trypsin inhibitor during all steps of chloroplast fractionation (Jackson *et al.*, 1998). Extraction of outer and inner plastid envelopes with 1 M NaCl or 0.1 M Na_2_CO_3_, pH 11, revealed that ^35^S-HPL and ^35^S-AOS were tightly bound to their respective target membranes (Fig. 2D). The localizations of HPL and AOS confirmed previous data for chloroplasts from Arabidopsis (Joyard *et al.*, 2010; Mwenda *et al.*, 2015) and tomato (Froehlich *et al.*, 2001), respectively, while not precluding divergent locations in other plant species (Farmaki *et al.*, 2007).

### Isolation of plastid envelope proteins interacting with AOS and HPL

We next attempted to identify proteins interacting with AOS in the envelope of Arabidopsis chloroplasts. C-terminal, (His)_6_-tagged AOS [AOS-(His)_6_] was expressed in bacteria and purified to apparent homogeneity by Ni-NTA chromatography. The chemically pure protein was incubated with isolated chloroplasts in standard import reactions containing 2.5 mM Mg-ATP (Froehlich *et al.*, 2001). After incubation, mixed outer and inner envelopes were isolated from ruptured chloroplasts (Li *et al.*, 1991; Schnell *et al.*, 1994) and solubilized with 1.3% decyl maltoside (Caliebe *et al.*, 1997). Proteins adhering to AOS-(His)_6_ were subjected to size exclusion chromatography and AOS-(His)_6_ was detected with AOS-specific or (His)_6_ antibodies (Laudert *et al.*, 1996; Froehlich *et al.*, 2001). For comparison, AOS-containing plastid envelope complexes were isolated from transgenic plants overexpressing AOS-FLAG under the control of the 35S cauliflower mosaic virus promoter.

Figure 3A and B depict the elution profiles of AOS after its purification from either isolated chloroplasts following the *in vitro* import reaction of AOS-(His)_6_ or transgenic plants overexpressing AOS-FLAG. Whereas AOS-FLAG and AOS-(His)_6_ were present in higher molecular mass complexes of ~250 kDa (peak I), a second, lower molecular mass complex was detectable only for AOS-FLAG (peak II). The lack of a corresponding AOS-(His)_6_ peak may be due to the lower abundance of this protein in the *in vitro* uptake assays with isolated chloroplasts. In control experiments using protein extracts from transgenic plants overexpressing HPL-FLAG, a single, low molecular mass peak was seen, the elution of which was virtually the same as that observed for AOS-FLAG in peak II (Supplementary Fig. S3). We assumed that the AOS-FLAG and HPL-FLAG contained in the low molecular mass peak represent enzyme monomers not interacting with other plastid envelope proteins. When the total pattern of proteins that co-purified with AOS-(His)_6_ in peak I was analyzed by SDS-PAGE, at least 10 bands were found (Fig. 3C, panel a, lane 1); of these, besides the bait protein, the ~100 kDa band was identified by protein sequencing as LOX2 and the ~72 kDa and ~24 kDa bands were identified as AOC2, forming SDS-resistant trimers and monomers, respectively. Western blotting using monospecific antibodies to Arabidopsis LOX2 (Supplementary Fig. S4) confirmed the identity of the ~100 kDa band as LOX2 (Fig. 3C, panel b). When similar experiments were carried out with HPL-(His)_6_, a completely different protein pattern was obtained, which, most notably, did not contain any of the proteins detected with AOS-(His)_6_ (Fig. 3C, panel a, lane 2).

### Genetic evidence for the existence of a chloroplast envelope complex comprising AOS, LOX2, and AOC2

**Fig. 4.**
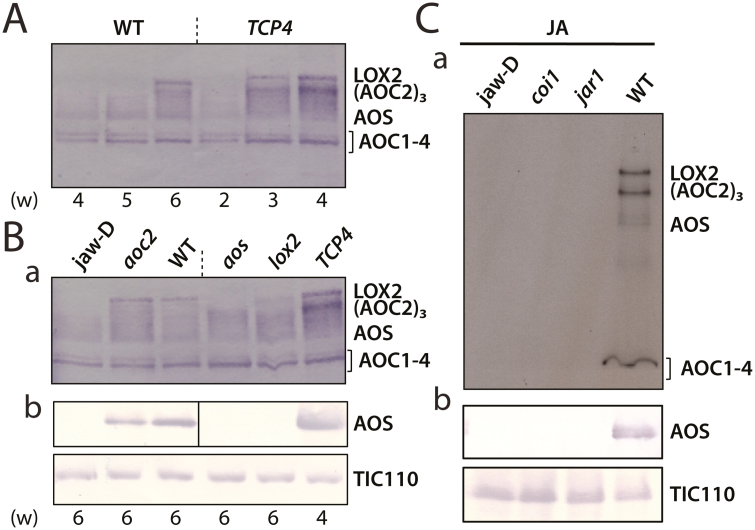
Genetic dissection of LOX2-AOS-AOC2 complex formation. (A) Time course, in weeks (w), of AOS, LOX2, and AOC2 accumulation in wild-type (WT) and *TCP4* plants expressing a mi319-resistant version of TCP4 during development. (B) Time course of accumulation of AOS, LOX2, and AOC in plants of *jaw-*D, *aoc2*, WT, *lox2*, *aos*, and *TCP4* backgrounds. (C) Accumulation of AOS, LOX2, and AOC in plastid envelopes of 4-week-old *jaw-*D, *coi1*, *jar1*, and WT plants after treatment with 45 µM MeJA for 24 h. For SDS-PAGE and western blotting, 40 µg (A, B) or 20 µg (C) of total plastid envelope protein was used. Western blots were simultaneously probed with antisera against LOX2, AOS, and AOC2 and developed with either horseradish peroxidase–alkaline phosphatase-based (A, panel a in B) or enhanced chemiluminescence-based (C) detection systems. For comparison, replicate blots were probed with monospecific antibodies to AOS and TIC110, respectively, and signals developed with an alkaline phosphatase-5-bromo-4-chloro-3-indolyl phosphate/nitro blue tetrazolium-based system (panel b in B and in C).

Because the expression of *LOX2*, *AOS*, and *AOC2* in Arabidopsis is under the control of the microRNA 319 (miR319)-regulated clade of *TCP* (*TEOSINTE BRANCHED/CYCLOIDEA/PCF*) transcription factor genes (Schommer *et al.*, 2008), we used the *jaw-*D mutant with strongly reduced mi319-dependent expression of JA biosynthetic genes to investigate the interaction of LOX2, AOS, and AOC2 genetically. When *jaw-*D plants, in which *TCP4* expression is strongly down-regulated, and primary transformants expressing a miR319-resistant version of *TCP4* (referred to herein as *TCP4* plants) that dominantly regulates *LOX2* expression (Palatnik *et al.*, 2003; Schommer *et al.*, 2008) were grown for 14 days in a greenhouse, not much difference in phenotype was seen. However, phenotypic differences were more pronounced at later stages of plant development and especially when plants matured and entered senescence. In agreement with previous results (Schommer *et al.*, 2008), *TCP4* plants displayed a marked acceleration of senescence, whereas *jaw-*D plants exhibited a considerable (2-week) delay in leaf senescence. These differential changes correlated with altered patterns of expression of the plastid envelope proteins LOX2, AOS, and AOC2 (Fig. 4; see also Supplementary Fig. S13, which shows respective densitometric analyses using ImageJ software, https://imagej.net/). *TCP4* plants more rapidly accumulated all three JA biosynthesis enzymes than wild-type plants (Fig. 4A).

JA biosynthesis is under the control of a feed-forward loop and consequently can be boosted by exogenously added JA (Wasternack and Hause, 2013; Yan *et al.*, 2013). To pinpoint the role of LOX2, AOS, and AOC2 in this loop, JA-deficient seedlings of the *aos* mutant (Park *et al.*, 2002; von Malek *et al.*, 2002) and JA-Ile-insensitive seedlings of the *coi1* mutant (Feys *et al.*, 1994) were used. For comparison, we used Arabidopsis mutants defective in the *LOX2* and *AOC2* genes, respectively (Seltmann *et al.*, 2010; Stenzel *et al.*, 2012). When the patterns of expression of plastid envelope proteins were compared, some differential effects were observed. As displayed in Fig. 4B, the *aoc2* knockout mutants contained wild-type levels of AOC1, AOC3, and AOC4, as well as wild-type levels of AOS and LOX2. By contrast, *aos* and *lox2* mutants expressed reduced amounts of LOX2 and AOC2 trimers, while retaining the wild-type levels of AOC1-4 monomers. These findings were suggestive of partially overlapping roles and expression patterns of the four AOC family members (see Supplementary Figs S5 and S6), with AOC1, AOC3, and AOC4 presumably being capable of replacing AOC2 and forming homo- and heterocomplexes. It has recently been demonstrated that all four AOCs display identical catalytic activities (Otto *et al.*, 2016).

To further dissect the feed-forward loop operating in JA biosynthesis, seedlings of the JA-insensitive *coi1* mutant (Feys *et al.*, 1994) and the JA-resistant *jar1* mutant (Staswick *et al.*, 1992) were used. *JAR1* encodes an enzyme that provides the physiologically active JA-Ile conjugate for CORONATINE INSENSITIVE 1 (COI1)-dependent signal transduction (Staswick *et al.*, 1992; Feys *et al.*, 1994). *coi1* and *jar1* seedlings were grown for 14 days in continuous white light and then sprayed with MeJA. MeJA treatment triggered the expression of LOX2, AOS, and AOC1-4 in wild-type plants but not in *jaw*-D, *coi1*, and *jar1* plants (Fig. 4C). Hence, JA production was required and relied on an intact JA signal transduction chain comprising JA-Ile and COI1, linking JA signal with miR319-regulated TCPs.

### Probing the interaction of LOX2, AOS, and AOC2 through cross-linking

The presence of higher molecular mass complexes containing LOX2, AOS, and AOC2 in the envelope of Arabidopsis chloroplasts suggested the possibility that the enzymes may interact physically and functionally in JA precursor biosynthesis. As a first step to test this hypothesis, cross-linking experiments were conducted using bacterially expressed, (His)_6_-tagged, purified precursors that had been derivatized with ^125^I-APDP, which is a heterobifunctional, photoactivatable, and cleavable label-transfer cross-linker frequently used in the chloroplast protein import field (e.g. Ma *et al.*, 1996). ^125^I-APDP contains a 21 Å spacer arm that, depending on its location on the bait protein, can penetrate to different extents into the respective target membrane and label proteins and lipids.

**Fig. 5.**
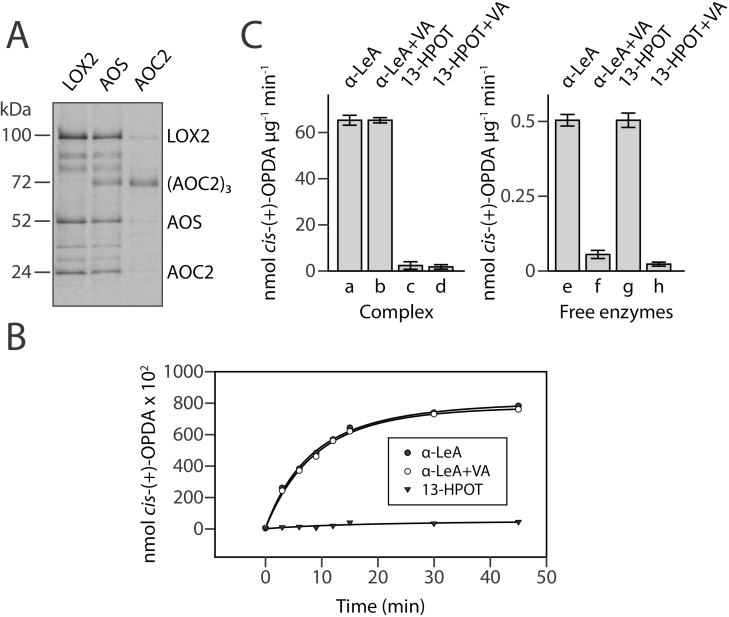
Channeling of jasmonate precursors in the LOX2-AOS-AOC2 plastid envelope complex. (A) Label-transfer cross-linking by ^125^I-APDP-LOX2, ^125^I-APDP-AOS, and ^125^I-APDP-AOC2 of proteins in mixed outer and inner envelopes of Arabidopsis chloroplasts. (B) Time course of *cis*-(+)-12-OPDA formation from α-LeA (filled circles) and 13-HPOT (filled triangles) by the isolated envelope complex containing LOX2, AOS, and AOC2. For comparison, *cis*-(+)-12-OPDA formation from α-LeA was tested in the presence of vernolic acid (VA) (open circles). Enzyme activities are expressed in nmol OPDA per µg^−1^ AOC2. (C) Rate of *cis*-(+)-12-OPDA formation determined after 10 min incubations for α-LeA (lanes a and e) and 13-HPOT (lanes c and g) by virtue of the LOX2-AOS-AOC2 envelope complex (lanes a–d), as compared to free enzymes released from the membrane complex by treatment with 3% SDS and dialyzed before analysis (lanes e–h). Respective controls show incubations performed with VA (lanes b, d, f, and h). Enzyme activities are expressed in nmol OPDA per min^−1^ µg^−1^ AOC2. Error bars refer to three independent experiments measured in triplicate.

^125^I-APDP-labeled LOX2, AOS, and AOC2 were imported into isolated Arabidopsis chloroplasts in darkness (Springer *et al*, 2016; Fig. 2, and Supplementary Fig. S7). After incubation, label-transfer cross-linking was induced by exposure to ultraviolet light with the chloroplasts on ice. Envelopes were then isolated from ruptured chloroplasts and solubilized with 3% SDS (Ma *et al.*, 1996), and ^125^I-APDP-labeled proteins were detected by SDS-PAGE and autoradiography. As shown in Fig. 5A, different banding patterns were revealed for the three labeled proteins. LOX2 and AOC2 (which was present as a trimer) both interacted with AOS, with little or no direct interaction between the two of them. For ^125^I-APDP-AOS, label transfer occurred on to LOX2 and AOC2 (Fig. 5A; see also the densitometric scans in Supplementary Fig. S13). Interestingly, ^125^I-APDP-AOS gave rise to both ^125^I-labeled AOC2 trimers and monomers (Fig. 5A). AOC2 monomers were also seen in assay mixtures containing ^125^I-APDP-LOX2. This observation could suggest a shuffling of monomers and trimers of AOC2 into the envelope complex. On the other hand, the nature of the chosen cross-linker and its topology on the bait protein may explain this result. Last but not least, it is also possible that substrate binding and conversion affected the interaction of LOX2, AOS, and AOC2, and thereby had an impact on the cross-linking results.

If LOX2, AOS, and AOC2 were to interact structurally and functionally, they could provide the possibility of substrate channeling without concurrent side reactions in *cis*-(+)-12-OPDA synthesis from α-LeA. To examine this possibility, bacterially expressed and purified, non-^125^I-APDP-derivatized AOS-(His)_6_ was imported into Arabidopsis chloroplasts. Then, the plastids were lysed and protein complexes containing AOS-(His)_6_ were isolated by affinity chromatography from 1.3% decyl maltoside-solubilized envelopes. For reference, SDS-dissociated and dialyzed envelope complexes containing free LOX2, AOS, and AOC2 in amounts identical to those in the intact complexes were used. Activity measurements using α-LeA and 13-HPOT revealed a tight channeling of metabolites in the intact envelope complex. This is apparent from time courses of *cis*-(+)-12-OPDA formation from α-LeA and 13-HPOT (Fig. 5B) and respective quantification of *cis*-(+)-12-OPDA (Fig. 5C). While α-LeA was converted to *cis*-(+)-12-OPDA by the isolated, intact (non-SDS-dissociated) plastid envelope complex, 13-HPOT was not accepted as a substrate. In line with the tight channeling of metabolites, the AOC inhibitor vernolic acid [(+/-)-cis-12,13-epoxy-9(*Z*)-octadecenoic acid] (Hofmann *et al.*, 2006) was unable to impede *cis*-(+)-12-OPDA synthesis (Fig. 5C). When applied to the SDS-dissociated, dialyzed complex containing the free, catalytically active LOX2, AOS, and AOC2 enzymes, however, vernolic acid did block *cis*-(+)-12-OPDA synthesis from α-LeA and 13-HPOT (Fig. 5C). Remarkably, the rate of *cis*-(+)-12-OPDA formation from α-LeA in the intact complex was 120-fold higher than that in the free-enzyme assay (Fig. 5C), suggesting that tight functional LOX2-AOS-AOC2 interactions occur and boost *cis*-(+)-12-OPDA synthesis. In summary, we obtained 2-fold higher activities for the free-enzyme assay as compared to Otto *et al.* (2016), that is, 0.5 nmol OPDA min^−1^ µg^−1^ AOC2 and a 240-fold greater activity for the complex-containing assay (60 nmol OPDA min^−1^ µg^−1^ AOC2).

### Interaction of LOX2, AOS, and AOC2 probed in the split-ubiquitin system

**Fig. 6.**
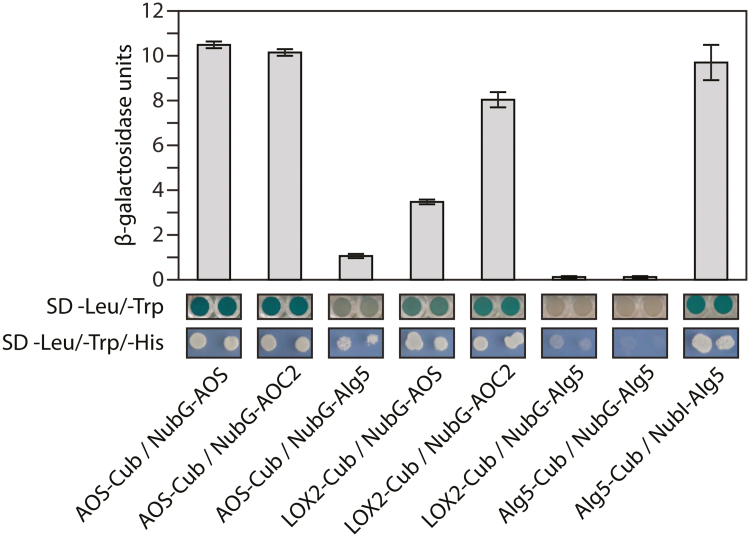
Genetic dissection of AOS-LOX2-AOX2 interactions in the split-ubiquitin system. Experiments were performed using fusions to the C-terminal (Cub) and N-terminal (NubG) halves of ubiquitin. Alg5-NubI, the fusion of the unrelated endoplasmic reticulum membrane protein Alg5 to NubI, was used as positive control. Negative controls were fusions of Alg5 to Cub (Alg5-Cub) or NubG (Alg5-NubG). Qualitative estimation of interactions was deduced from the growth behavior of co-transformed yeast cells on solidified minimal synthetic defined (SD) base medium supplemented with -Leu/-Trp/-His dropout supplements (Clonetech). Yeast strains were plated to an OD_600_ of 0.8 (left colony) and at 1:1 dilution (right colony). For quantitative assays the β-galactosidase activity of cells grown in SD liquid culture containing -Leu/-Trp dropout supplements was analyzed.

As an independent approach to confirm the interaction of LOX2, AOS, and AOC2, we carried out split-ubiquitin yeast two-hybrid screens for membrane-bound proteins. Yeast cells (strain DSY1) were first transformed with AOS- and LOX2-containing bait vectors (AOS-Cub, LOX2-Cub), which express the bait proteins fused to the C-terminal part of ubiquitin. For each bait protein, we constructed and tested three different vectors (pAMBV4, pCMBV4, and pTMBV4), providing promoters of different strength to drive bait protein expression. In our experiment, the pTMBV4 vector, which contains a highly potent TEF1 promoter, gave the clearest results. Co-transformation was accomplished in a second, separate step in which AOS- and AOC2-containing prey vectors were introduced into the selected yeast cells (NubG-AOS, NubG-AOC2). As prey vectors, we used constructs that added the N-terminal part of ubiquitin to the N-terminal extremity of the prey proteins (pADSL-Nx). In addition to the specific constructs used to study potential LOX2-AOS-AOC2-interactions, we used control vectors provided with the system to test for non-specific activation and autoactivation (NubG-Alg5), as well as positive (Alg5-Cub, NubI-Alg5) and negative (Alg5-Cub, NubG-Alg5) system controls. The co-transformed yeast cells were subjected to qualitative His-complementation growth tests carried out on SD plates supplemented with 5 mM 3-amino-1,2,4-triazole and lacking the amino acids Leu, Trp, and His. Additionally, the relative β-galactosidase activities of the investigated co-transformed yeast strains were determined to provide quantitative data on the strength of interaction of the analyzed protein pairs. According to the results summarized in Fig. 6, interactions were observed between AOS and AOC2, with a fraction of AOS presumably forming dimers, as found for AOS from *Parthenium argentatum* (Chang *et al.*, 2008; Li *et al.*, 2008). Some interactions could also be detected for LOX2 with AOC2 and AOS, although the strengths of these interactions were dependent on the respective bait and prey protein and presumably reflected the different membrane compositions of yeast cells versus higher plant chloroplast envelopes.

## Discussion

### Sequestration of jasmonate precursor biosynthesis through a protein complex in the plastid envelope

In the present study, evidence is provided for the existence of a protein complex involved in JA precursor biosynthesis in Arabidopsis chloroplasts. We show that LOX2, AOS, and AOC2, the enzymes that catalyze consecutive steps in JA precursor biosynthesis (Fig. 1), are co-localized in the inner envelope of Arabidopsis chloroplasts (Fig. 2 and Supplementary Fig. S7; see also Springer *et al.*, 2016), and form complexes operating in *cis-*(+)-12-OPDA synthesis from α-LeA (Figs 3 and 5; Supplementary Fig. S8). It was possible to reconstitute a similar complex *in vitro* from soluble, catalytically active LOX2, AOS, and AOC2 enzymes and isolated plastid envelope lipids (Supplementary Fig. S8). In either case, LOX2, AOS, and AOC2 were present in a 1:1:4 stoichiometry, indicating that these complexes may contain both AOC2 monomers and trimers. The significance of this observation and the possibility of activity regulation through heterotrimerization of AOC2 with AOC1, 3, and 4 remains to be established. It must be noted in this context, however, that Zerbe *et al.* (2007) also used AOS and AOC2 at a 1:4 molar ratio. Interaction studies in the split-ubiquitin yeast two-hybrid system confirmed the interaction of AOS and AOC2 (Fig. 6). When isolated plastid envelope complexes were supplied with α-LeA, only *cis*-(+)-12-OPDA accumulated in the reaction medium (Fig. 5). No evidence was obtained for the release of significant amounts of HPOT or EOT and its short-lived disintegration products (α-ketols and γ-ketols), as well as of racemic OPDA, into the reaction mixture. These findings are suggestive of a tight channeling of metabolites through both the isolated and the reconstituted protein complexes. By virtue of the observed channeling of metabolites, the dilution of the reaction intermediates was kept low. On the other hand, the observed channeling of metabolites greatly increased the rate of formation and, thus, the yield of *cis-*(+)-12-OPDA from α-LeA. Compared with the free-enzyme assay, 120-fold higher activities were measured in the isolated envelope complex. Our data confirm and extend previous findings by Zerbe *et al.* (2007), who reconstituted *cis-*(+)-12-OPDA synthesis from 13-HPOT by combining purified recombinant AOS and AOC2 *in vitro*. The authors found that both soluble and matrix-bound enzymes are active and that their co-fixation on a solid matrix increased the yield of *cis-*(+)-OPDA from 13-HPOT by ~50%. In contrast to those studies, however, in which AOS and AOC2 were randomly bound to the matrix, thus excluding tight substrate channeling, in our experiments the enzymes were associated in an orderly fashion, thereby allowing the channeling of α-LeA. Because vernolic acid failed to inhibit *cis-*(+)-12-OPDA synthesis from α-LeA or 13-HPOT in the native and reconstituted complexes (Fig. 5; Supplementary Fig. S8), we conclude that the active site of AOC2 was largely inaccessible to the inhibitor. On the other hand, exogenously administered 13-HPOT was not accepted as substrate for *cis-*(+)-12-OPDA synthesis by the native and reconstituted complexes, but it was accepted in the free-enzyme assay (Fig. 5C). On the basis of these results we conclude that synthesis of *cis-*(+)-12-OPDA from α-LeA is strictly compartmentalized, presumably to prevent competing side reactions, such as that from 13-HPOT catalyzed by HPL or by chemical decay, which give rise to α- and γ-ketols. By this means, plants avoid the costly formation of *n*-hexenal and 12-oxo acids implicated in direct and indirect defenses against herbivores (Wu and Baldwin, 2010; Hoffmann *et al.*, 2011), while maintaining the capacity to respond to biotic challenges and abiotic stresses (Wasternack and Hause, 2013; Yan *et al.*, 2013).

### Structural modeling of the LOX2-AOS-AOC2 envelope complex

**Fig. 7.**
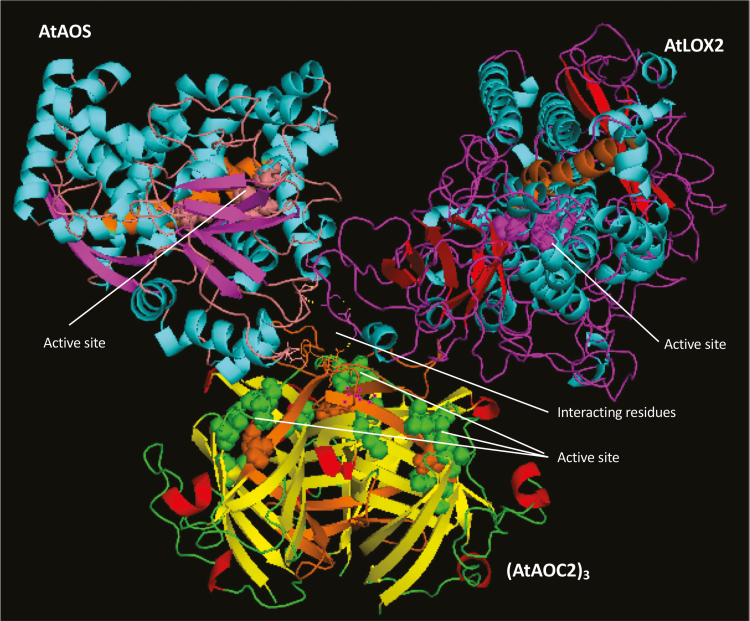
Structural model of the LOX2-AOS-(AOC2)_3_ interaction in the chloroplast inner envelope. The transmembrane domain is shown in orange. Active sites are shown with spheres and interacting residues with lines. Active site predictions for LOX2 are based on Youn *et al.* (2006), for AOS2 on Lee *et al.* (2008), and for AOC2 on Hofmann *et al.* (2006), and transmembrane domain predications are based on TMpred for LOX2 and (AOC2)_3_.

We conclude from our results that AOS forms complexes with both LOX2 and AOC2 *in planta* as well as *in vitro*. Molecular modeling was carried out to obtain insight into the potential structure of this complex, using published X-ray structure data for soybean lipoxygenase L3 (PDB ID 1LNH; Skrzypczak-Jankun *et al.*, 1997; Youn *et al.*, 2006), AOS (D 3CLI; Lee *et al.*, 2008), and AOC2 (PDB ID 2GIN; Hofmann *et al.*, 2006), obtained at 2.60 Å, 1.80 Å, and 1.80 Å resolution, respectively, as templates (Supplementary Figs S9–S11). First, homology modeling was done for LOX2, based on the X-ray structure of soybean LOX L3 (Cho and Stahelin, 2006; Youn *et al.*, 2006). The established LOX2 structure of Arabidopsis (Supplementary Fig. S9) was then used in modelling of the whole LOX2-, AOS-, and AOC2-containing complex. The top rank model (depicted in Fig. 7) suggests that at least two amino acid residues, Ser92 and Gly94, of LOX2 are potentially involved in the LOX2-AOS interaction, forming hydrogen bonds with Ser272 of AOS (Supplementary Fig. S12A). The interacting Ser and Gly residues of LOX2 are not conserved between LOX2 and soybean LOX3 and reside in a loop region between β-sheets 1 and 2 of LOX2. Similarly, one region of interacting amino acids could be identified for each of the hypothetical LOX2-AOC2 and AOS-AOC2 complexes. In these complexes, Asp96 of LOX2 is predicted to interact with Asn42 of AOC2, and Phe44 and Ser45 of AOC2 were found when analyzing these enzymes apart from LOX2, while they were not seen in the modeled LOX2-AOS-AOC2 complex (Supplementary Fig. S12B).

Details of how the LOX2-AOS-AOC2 complex may be bound to the membrane remain elusive. The fact that a functional complex could be reconstituted from soluble LOX2, AOS, and AOC2 molecules at first glance suggests that the lipid bilayers might not be required for the formation of the whole complex. Nevertheless, all three enzymes attained salt- and protease-resistant states after respective *in vitro* import reactions with chloroplasts, suggesting that they are tightly bound to the lipid bilayers of the inner plastid envelope. Hydrophobic transmembrane domains may anchor LOX2, AOS, and AOC2 in the membrane. Lee *et al.* (2008) identified several non-polar detergent-binding α-helices in AOS, of which those designated α-F and α-G were tentatively defined as putative transmembrane domains in our structural models (Supplementary Fig. S10A). Predictions made with TMpredn, however, suggest that a different part of the AOS polypeptide forms a single transmembrane domain and that this overlaps with some of the active site residues (Supplementary Fig. S10B). Such an overlap would resemble that in the AOC2 trimer, where some active site residues appear to be embedded into, or are part of, the predicted transmembrane domain (Supplementary Fig. S11). However, it cannot be excluded that some of the hydrophobic β-sheets forming the characteristic β-barrel cavity are involved in membrane binding (Supplementary Fig. S11). Lastly, the LOX structure of Skrzypczak-Jankun *et al.* (1997) used for modelling LOX2 from Arabidopsis (Supplementary Fig. S9) suggests a hydrophobic environment of the catalytic pocket that could overlap with the predicted transmembrane domain. Further work is needed to resolve the structure of the LOX2-AOS-AOC2 complex and elucidate its membrane binding.

AOC2 and the other members of the AOC family easily form homo- and heterotrimers (Hofmann *et al.*, 2006; Hofmann and Pollmann, 2008; Otto *et al.*, 2016). As described by Otto *et al.* (2016), trimerization of AOC isoenzymes is likely to contribute to regulation of their activity. Several salt bridges between monomers and a hydrophobic core within the AOC2 trimer were identified and functionally proven by site-directed mutagenesis. While Lys152 of one monomer and Glu128 of the neighboring monomer established the observed salt bridges, the amino acids involved in building the hydrophobic core of the trimer comprised Leu40, Leu50, Leu53, and Ile79 of all three monomers (Otto *et al.*, 2016). Notable are the conservation of interacting amino acids and the overlapping expression patterns of AOC1/2 during germination, leaf production, rosette growth, inflorescence emergence, and flowering, both with each other and with the respective expression patterns of LOX2 and AOS (Supplementary Figs S4–6). By contrast, the expression pattern of AOC3 and AOC4 is more distinct (Supplementary Figs S4–6). On the other hand, HPL expression is comparably low in all of the developmental stages analyzed (Supplementary Fig. S6). Together, these data are suggestive of a co-evolution of mechanisms that allow JA precursor biosynthesis to be favored over the production of volatile compounds during plant development. This situation obviously changes when plants are challenged by chewing insects and both direct and indirect defenses are triggered through the HPL pathway (Bate and Rothstein, 1998; Blée, 1998; Croft *et al.*, 1993; Wu and Baldwin, 2010). In functional terms, both AOS and HPL belong to the same superfamily of non-canonical cytochrome P450 enzymes, but their amino acid sequences are distinct enough to permit their unique reaction mechanisms (Lee *et al.*, 2008). While AOS interacts with LOX2 and AOC2, no interacting partners could be identified for HPL (Fig. 3). Finally, the chloroplast localization of AOS and HPL is quite distinct and helps to assure that AOS operates in the channeling of α-LeA to OPDA, whereas HPL drives volatile production.

In agreement with previous publications (Spivey and Ovádi, 1999; Gilbert *et al.*, 2008), we conclude that compartmentalization of enzymes as well as organization into multi-protein complexes provides a highly specific cellular mechanism for controlling the flow of metabolites through key regulatory pathways and preventing unfavorable competing reactions. Work is in progress to obtain X-ray structural data for the identified LOX2-AOS-AOC2 complex from higher plant chloroplasts.

## Supplementary data

Supplementary data are available at *JXB* online.

Protocol S1. Supplementary experimental procedures.

Table S1. Primer sequences.

Fig. S1. Western blot analysis on the cross-reactivity of the AOS and HPL antibodies.

Fig. S2. Localization of AOS and HPL *in planta*.

Fig. S3. Size exclusion chromatography of AOS-FLAG and HPL-FLAG containing complexes.

Fig. S4. Identification of monospecific α-LOX2 antibodies.

Fig. S5. Amino acid sequence alignment of Arabidopsis AOC1-4.

Fig. S6. Developmental expression pattern of *HPL*, *LOX2*; *AOS*, *AOC1/2*, *AOC3*, and *AOC4*.

Fig. S7. Import to and localization of ^35^S-labelled AOC2 in isolated Arabidopsis chloroplasts.

Fig. S8. Reconstitution of LOX2-AOS-AOC2 complexes.

Fig. S9. Structural model of Arabidopsis LOX2.

Fig. S10. 3D model of Arabidopsis AOS.

Fig. S11. 3D model of the AOC2 trimer from Arabidopsis.

Fig. S12. Identification of amino acid residues involved in the formation of the predicted LOX2-AOS-AOC2 complex.

Fig. S13. Densitometric image analysis of the data presented in Figs 4 and 5.

Supplementary Table S1 and Figures S1-S7

Supplementary Figures S8-S12

Supplementary Dataset S1
