# Supplementary material for: Substrate channeling in oxylipin biosynthesis through a protein complex in the plastid envelope of Arabidopsis thaliana
Source: J Exp Bot. 2019 Jan 23;70(5):1483–95. doi: 10.1093/jxb/erz015 (PMC6411374; doi:10.1093/jxb/erz015)
Supplement: Supplementary Table S1 and Figures S1-S7 [file erz015_suppl_supplementary_table_s1_figures_s1-s7.pdf]

Supplementary information

Supplementary Experimental Procedures

cDNA Clones

cDNA clones of LOX2 (Bell et al., 1995; Bell and Mullet, 1993), AOS (Laudert et al., 1996), AOC2 (Stenzel et al., 2003; Schaller et al., 2008) and HPL (Froehlich et al., 2001) were cloned into appropriate vectors, allowing for coupled in vitro transcription/translation in rabbit reticulocyte lysates (LOX) and wheat germ extracts (AOS, HPL, AOC2), respectively, and bacterial expression (Innis et al., 1990).

Reconstitution of LOX2, AOS and AOC2-containing envelope lipid complexes

Amino-terminally or carboxy-terminally His-tagged or Flag-tagged version of LOX2, AOS and AOC2 (Otto et al., 2016; Springer et al., 2016; Zerbe et al., 2007) were expressed in bacteria and purified by Ni-NTA agarose affinity chromatography. Chemically pure enzymes (0.1 mg of protein each) were mixed in 10 mM PPi buffer, pH 7.0, and incubated for 1 h at 23 °C. The reaction mixtures were then loaded on a Sephadex G100 mini-column and centrifuged at 1000 *g* for 5 min to separate protein assemblies of LOX2, AOS and AOC2 running in the flow-through from unassembled subunits (free LOX2, AOS and AOC2) being retained on the column. In the recovered higher molecular mass complexes, LOX2, AOS and AOC2 were present at ≈10 µg, ≈5.2 µg and ≈9.4 µg, respectively. This corresponded to a LOX2:AOS:AOC2=1:1:4 stoichiometry. The complexes (1.5 nM final concentration) were supplemented with a plastid envelope lipid fraction that had been prepared from *Arabidopsis* chloroplasts (Sarkis et al., 2014). Briefly, total membranes were prepared from ruptured *Arabidopsis* chloroplasts and separated into thylakoids and envelopes (Douce and Joyard, 1982; Li et al., 1991). Membranes were dissolved at 5 mg protein/mL in a buffer containing 10 mM MOPS [3-(*N*-morpholino) propanesulfonic acid; pH 7.8], 1 mM phenylmethanesulfonyl fluoride, 1 mM benzamidine, and 5 mM caproic acid (Sarkis et al., 2014). Lipids then were extracted as described (Bligh and Dyer, 1959) and separated on silica gel columns (SIL-B-200; Sigma-Aldrich, St. Louis, MO, USA). Mixed galactolipids (MGDG and DGDG) were eluted with acetone, whereas phospholipids were eluted with a mixture of chloroform/methanol (50/50; v/v). MGDG, DGDG, and PG were purified further on thin-layer chromatography plates (silica gel 60; Merck-Millipore) with chloroform/acetone/methanol/acetic acid/water (50/20/10/10/5; v/v) and eluted as described (Bligh and Dyer Bligh, 1959). Lipids quantification was according to Jouhet et al. (2003). For reconstitution experiments, native envelope lipid mixtures or biomimetic lipid mixtures composed of MGDG:DGDG:PG (64/28/8) were used (Sarkis et al., 2014). The established lipid-containing higher molecular mass complexes were subjected to non-denaturing PAGE and protein complexes were identified by Western blotting using His- or Flag-tag antibodies. Peak fractions corresponding to those in Fig. 3 (peaks 5-7) were pooled and used for subsequent enzyme assays.

Immunoprecipitation of LOX2 with specific antibodies

Immunoprecipitation was conducted out in buffer A containing 10 mM Tris/HC1, pH 7.5, 0.15 M NaCl, 1 mM EDTA, 1% Triton X-100 and 0.1% SDS (Wiedmann et al., 1987) that had been supplemented with T7-RNA-polymerase (2 µg mL^{-1}, 100 kDa) to reduce unspecific LOX2 degradation. After incubation of chloroplast protein extract with the affinity-purified LOX2 antiserum at room temperature for 2 h and overnight at 4°C, 50 µl of a 30% suspension of protein A-Sepharose

(Pharmacia Fine Chemicals, Uppsala, Sweden) were added and the mixture was shaken for 20 min at 4°C. The Sepharose was washed five times with buffer A and the bound material eluted by boiling for 5 min in SDS gel sample buffer (Laemmli, 1970). The ≈100 kDa ghost band seen in Fig. S4 thus is due to the presence of the T7-RNA-polymerase carrier protein added during all steps of the immunoprecipitation procedure.

Production of transgenic lines expressing AOS with COOH-terminally $(His)_6$ or FLAG tags

Gateway technology was employed for the construction of transgenes for *in planta* transformation (Invitrogen). For the generation of transgenic 35S::AtAOS plants, a cDNA for the coding frame of AOS was amplified PCR and introduced into pDONR221 vector and, after sequencing, introduced into pB7FWG2 (Plant System Biology, VIB-Ghent University). For the construction of transgenic lines expressing 35S::AtAOS-$(His)_6$, a similar strategy was used, although the final vectors were modified to contain the coding region for the His tag (Boex et al., 2015). For the production of transgenic plants expressing COOH-terminally FLAG tagged AOS, a pEarleyGate 302-derived binary vector construct was used, allowing replication in both *Escherichia coli* and *Agrobacterium tumefaciens* and having left border (LB) and right border (RB) sequences for *Agrobacterium*-mediated T-DNA transfer. The pEarleyGate 302 cassette includes attR1, a chloramphenicol resistance gene (CmR), the ccdB killer gene and attR2. In addition, pEarleyGate 302 contains the cauliflower mosaic virus 35S promoter and its upstream enhancer, the 3' sequences of the octopine synthase gene, including polyadenylation and presumptive transcription termination sequences, the Basta herbicide resistance gene for selection of transgenic plants, as well as the FLAG DNA for affinity purification of tagged protein from plants (Earley et al., 2006).

Cytolocalization of AOS-Green Fluorescent Protein (GFP) and HPL-GFP in planta

Transgenic plants expressing AOS with COOH-terminally-tagged GFP were generated as described by Samol et al. (2011). Similarly, transgenic plants were ctreated expressing a HPL-GFP fusion protein. For localization studies, 4 weeks-old plants were used and subjected to confocal laser scanning microcopy. For the detection of GFP fluorescence, the excitation wavelength was 488 nm and the barrier filter BP 530 (band pass, 515–545 nm) was used. For monitoring chlorophyll fluorescence, the excitation wavelength was 568 nm and the barrier filter BP 590 (long pass, >590 nm) was used (Caroca et al., 2013). As controls, transgenic plants expressed ferredoxin (FD)-GFP or GFP alone were used (Rossig et al., 2013).

Split-Ubiquitin Yeast 2-Hybrid assay

In the yeast interaction assay, truncated cDNA versions of the corresponding genes have been used, lacking the coding sequences of their predicted signal peptides. The fragments Δ32*AOS* and Δ77*AOC2* have been constructed according to Laudert *et al.* (1998) and Zerbe *et al.* (2007), respectively. Transit peptides have further been confirmed by *in silico*-prediction using the ChloroP 1.1 server (Emanuelsson et al., 1999). The cDNA fragment of mature Δ32*AOS* lacking its stop codon has been amplified by PCR adding *Xba*I and *Nco*I restriction sites to the 5' and 3'-end, respectively. The same sites have been used to integrate the cDNA fragment into the bait vectors pTMBV4, pAMBV4 and pCMBV4. In case of *LOX2*, a Δ56*LOX2* cDNA fragment without stop codon was amplified by PCR, extending the coding region by additional *Spe*I and *Dra*I sites. The resulting fragment has been introduced into the bait vectors using the *Stu*I / *Xba*I sites included in the multiple cloning sites of the

vectors. In order to generate the *AOS* and *AOC2* containing prey vectors, the truncated cDNA fragments Δ32*AOS* and Δ77*AOC2* were PCR amplified adding *Sma*I / *Sal*I and *Bam*HI / *Sal*I restriction sites to their extremes, respectively. The same sites have been used to clone the two fragments into the multiple cloning site of the prey vector pADSL-Nx. All cDNA fragments obtained by PCR have been tested for sequence integrity by commercial sequencing prior to their integration into either bait or prey vectors. The primer pairs have been used for the construction of the vectors is given in table S1. Yeast transformation and activity assessment has been conducted in accordance to the manufacturer's protocol.

Molecular Modeling

3D-modeling of LOX2-AOS-AOC2 complexes was carried out, using ClusPro, an online protein structure and function prediction tool (Comeau et al., 2004). Published templates included the X-ray structures of LOX2 (Skrzypczak-Jankun et al., 1997), AOC2 (Hofmann et al., 2006) and AOS (Lee et al., 2008). The homology model of LOX was constructed using SWISS-MODEL (Schwede et al., 2003), with soybean lipoxygenase-3 (PDB ID: 1LNH) as template, yielding a model with a global quality estimation score (GMQE) of 0.75.

References

Bell E, Mullet JE (1993). Characterization of an Arabidopsis lipoxygenase gene responsive to methyl jasmonate and wounding. Plant Physiol 103:1133-1137.

Bell E, Creelman RA, Mullet JE (1995). A chloroplast lipoxygenase is required for wound-induced jasmonic acid accumulation in Arabidopsis. Proc Natl Acad Sci U S A 92:8675-8679.

Bligh EG, Dyer WJ (1959) A rapid method of total lipid extraction and purification. Can. J. Biochem. Physiol 37, 911–917

Boex-Fontvieille E, Rustgi S, von Wettstein D, Reinbothe S, Reinbothe C (2015). Water-soluble chlorophyll protein is involved in herbivore resistance activation during greening of Arabidopsis thaliana. Proc Natl Acad Sci USA 112(23):7303-738.

Caroca R, Howell KA, Hasse C, Ruf S, Bock R, 2013. Design of chimeric expression elements that confer high-level gene activity in chromoplasts. Plant J 73(3): 368-379.

Comeau SR, Gatchell DW, Vajda S, Camacho CJ (2004) ClusPro: an automated docking and discrimination method for the prediction of protein complexesBioinformatics. 20(1):45-50.

Douce R., Joyard J (1982) Purification of the chloroplast envelope. In Methods in Chloroplast Molecular Biology (Edelman M., Hallick R., Chua N.-H. , eds) pp. 239–256, Elsevier, Amsterdam

Earley KW, Haag JR, Pontes O, Opper K, Juehne T, Song K, Pikaard CS 2006. ateway-compatible vectors for plant functional genomics and proteomics. Plant J 45(4), 616-629.

Emanuelsson O, Nielsen H, von Heijne G (1999) ChloroP, a neural network-based method for predicting chloroplast transit peptides and their cleavage sites. Protein Sci 8(5):978-84.

Froehlich JE, Itoh A, and Howe GA (2001). Tomato allene oxide synthase and fatty acid hydroperoxide lyase, two cytochrome P450s involved in oxylipin metabolism, are targeted to different membranes of chloroplast envelope. Plant Physiol 125:306-317.

Innis MA, Gelfand DH, Sninsky JJ, White TJ (1990). PCR protocols: a guide to methods and applications. San Diego, CA, USA: Academic Press.

Jouhet J, Maréchal E, Bligny R, Joyard J, Block MA (2003) Transient increase of phosphatidylcholine in plant cells in response to phosphate deprivation. FEBS Lett 544, 63–68

Laemmli UK (1970) Cleavage of structural proteins during the assembly of the head of bacteriophage T4.Nature 227(5259): 680-685.

Laudert D, Pfannschmidt U, Lottspeich F, Holländer-Czytko H, Weiler EW (1996). Cloning, molecular and functional characterization of *Arabidopsis thaliana* allene oxide synthase (CYP 74), the first enzyme of the octadecanoid pathway to jasmonates. Plant Mol Biol 31:323-335.

Lee DS, Nioche P, Hamberg M, Raman CS (2008) Structural insights into the evolutionary paths of oxylipin biosynthetic enzymes. Nature 455(7211):363-368.

Li HM, Moore T, Keegstra K (1991) Targeting of proteins to the outer envelope membrane uses a different pathway than transport into chloroplasts. Plant Cell 3(7):709-17

Otto M, Naumann C, Brandt W, Wasternack C, Hause B (2016) Activity regulation by heteromerization of Arabidopsis allene oxide cyclase family members. Plants (Basel5(1). pii: E3.

Rossig C, Reinbothe C, Gray J, Valdes O, von Wettstein D, Reinbothe S. 2013. Three proteins mediate import of transit sequence-less precursors into the inner envelope of chloroplasts in Arabidopsis thaliana. Proc Natl Acad Sci USA 110(49):19962-19967.

Samol I, Rossig C, Buhr F, Springer A, Pollmann S, Lahroussi A, von Wettstein D, Reinbothe C, Reinbothe S 2011. The outer chloroplast envelope protein OEP16-1 for plastid import of NADPH:protochlorophyllide oxidoreductase A in Arabidopsis thaliana. Plant Cell Physiol 52(1):96-111.

Sarkis J Rocha J Maniti O Jouhet J, Vié V, Block MA, Breton C, Maréchal E, Girard-Egrot A (2014) The influence of lipids on MGD1 membrane binding highlights novel mechanisms for galactolipid biosynthesis regulation in chloroplasts. FASEB J. 2014 Jul;28(7):3114-23.

Schaller F, Zerbe P, Reinbothe S, Reinbothe C, Hofmann E, Pollmann S (2008). The allene oxide cyclase family of *Arabidopsis thaliana*: localization and cyclization. FEBS J 275:2428-2441.

Schwede T, Kopp J, Guex N, Peitsch MC (2003) SWISS-MODEL: An automated protein homology-modeling server. Nucleic Acids Res 31(13):3381-5.

Skrzypczak-Jankun E, Amzel LM, Kroa BA, Funk MO (1997) Structure of soybean lipoxygenase L3 and a comparison with its L1 isoenzyme. Proteins: Structure Function ad Genetics 29:15–31.

Springer A, Kang C, Rustgi S, von Wettstein D, Reinbothe C, Pollmann S, Reinbothe S (2016) rogrammed chloroplast destruction during leaf senescence involves 13-lipoxygenase (13-LOX). Proc Natl Acad Sci USA 113(12):3383-8.

Stenzel I, Hause B, Miersch O, Kurz T, Maucher H, Weichert H, Ziegler J, Feussner I, Wasternack C (2003). Jasmonate biosynthesis and the allene oxide cyclase family of Arabidopsis thaliana. Plant Mol Biol 51:895-911.

Wiedmann M, Kurzchalia TV, Bielka H, Rapoport TA (1987) Direct probing of the interaction between the signal sequence of nascent preprolactin and the signal recognition particle by specific cross-linking. J Cell Biol 104(2): 201-208.

Zerbe P (2007) Molekulare Analyse der Biosynthese octadecanoid-abgeleiteter Signalmoleküle durch Allenoxid-Synthase und Allenoxid-Cyclase aus *Arabidopsis thaliana* (L.) HEYNH. Dissertation, http://www-brs.ub.ruhr-uni-bochum.de/netahtml/HSS/Diss/ZerbePhilipp/diss.pdf, Ruhr-University Bochum, Germany

Zerbe P, Weiler EW, Schaller F (2007) Preparative enzymatic solid phase synthesis of *cis*(+)-12-oxo-phytodienoic acid - physical interaction of AOS and AOC is not necessary. Phytochemistry 68(2):229-236.

Zimmermann P, Hirsch-Hoffmann M, Hennig L, Gruissem W (2004) GENEVESTIGATOR. Arabidopsis Microarray Database and Analysis Toolbox. Plant Physiol 136(1):2621-2632.

Table S1. Primer sequences used for split-ubiquitin yeast 2-hybrid screens

Name	Sequence 5'-3'
Bait vectors:	
AOS-Δ32-*Xba*I-ATG-For	TATTCTAGAAAAATGCCGGGTCAGAAACTCC
AOS-Δ32-*Nco*I-Rev	TATCCCATGGAGAAAGCTAGCTTTCCTTAACGAC
LOX2-Δ56-*Spe*I-For	TATACTAGTAAAAATGGCTAATATTGAACAAGAAGGTA
LOX2-Δ56-*Dra*I-Rev	TATTTTAAAAGAATAGAAATACTATAAGGAACACC
Additional primer for sequencing of Δ56*LOX2*:	
LOX2-650-Seq	ATCCAAGGCGTGCAAGACC
LOX2-1500-Seq	AGGACTCATGCCTGTACGG
Prey vectors:	
AOS-Δ32-*Sma*I-Nx-For	TATCCCGGGAAGCTTCCGGGTCAGAAACTC
AOS-Δ32-STOP-Nx-*Sal*I-Rev	TATGTCGACCTAAAAGCTAGCTTTCCTTAAC
AOC2-Δ77-*Bam*HI-START-Nx	TATGGATCCCCAAGCAAAGTTCAAGAAGAACTG
AOC2-Δ77-SalI-Nx-Rev	TATATCGATTTAGTTGGTATAGTTACTTATAAC

Supplementary Figures

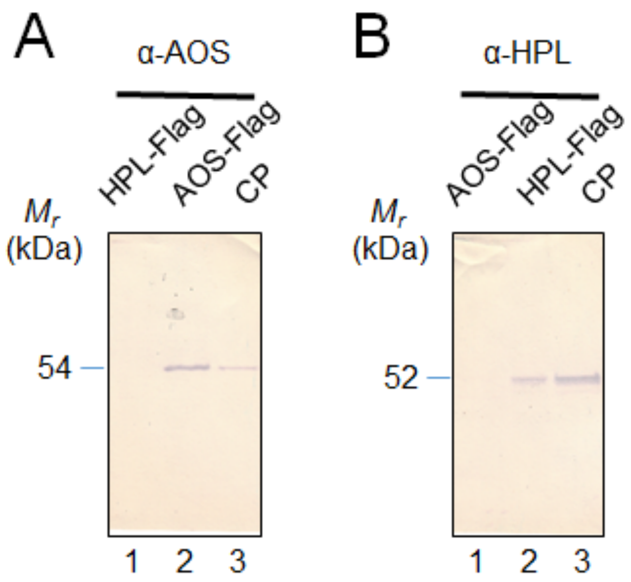

Fig. S1. Lack of cross-reactivity of the AOS and HPL antibodies. Mono-specific antibodies were raised against AOS (**A**) and HPL (**B**) and tested for their cross-reactivity with each other, using bacterially expressed AOS-Flag and HPL-Flag (2 µg of protein each) as well as chloroplast envelope (CP) protein extracts (40 µg of protein). Protein detection was made with an alkaline phosphatase- 5-Bromo-4-chloro-3-indolyl phosphate/nitro blue tetrazolium (NBT)-based system. Sizes of the detected protein bands are indicated.

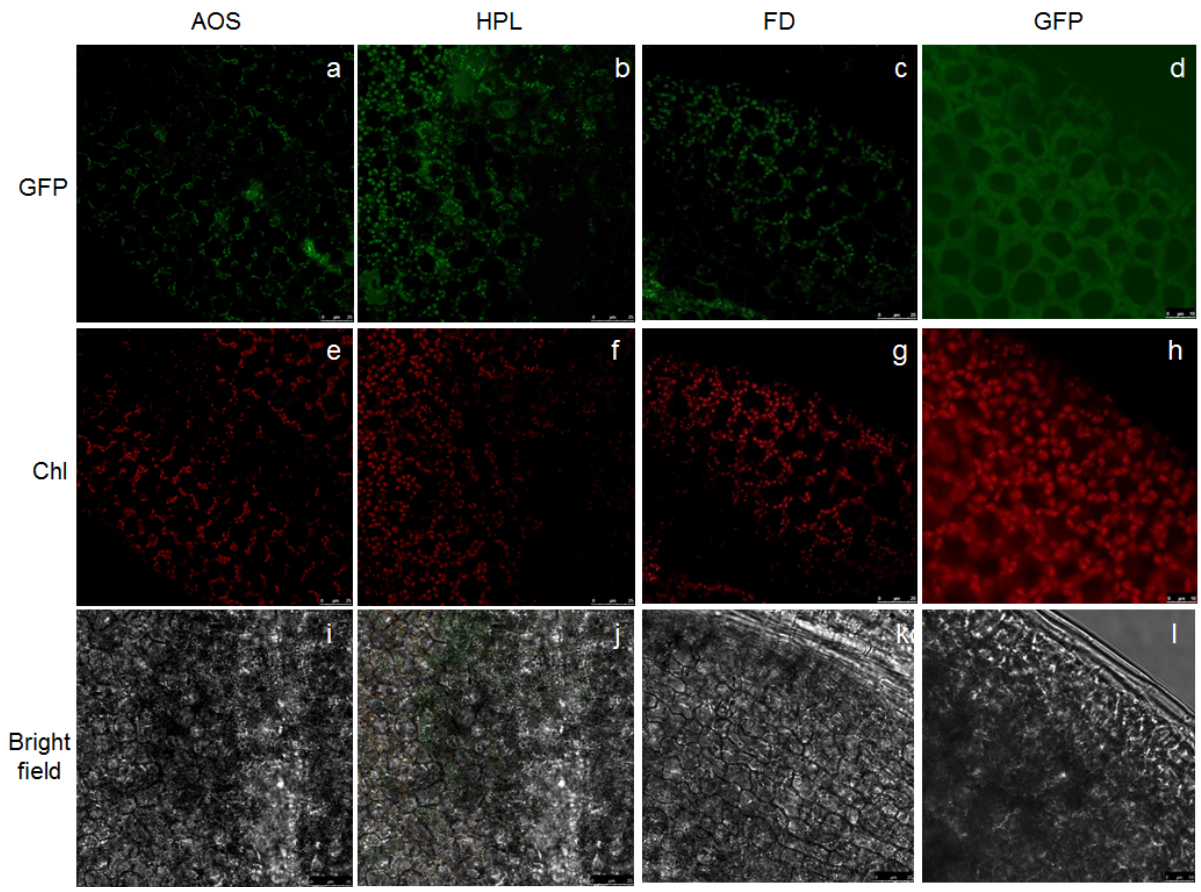

Figure S2. Localization of AOS and HPL in planta. Transgenic plants were generated expressing AOS or HPL and COOH-terminally tagged green fluorescent protein (GFP) and analyzed by confocal laser scanning microscopy. For comparison, transgenic plants were used expressing a fusion protein consisting of ferredoxin (FD) fused to GFP or GFP alone without any fusion protein attached to it. The images show GFP fluorescence (panels a-d), chlorophyll autofluorescence (panels e-h) and respective bright field analyses (panels i-l) for AOS-GFP plants (panels a, e and i), HPL-GFP plants (panels b, f and j), FD-GFP plants (panels c, g and k) and GFP plants (panels d, h and l).

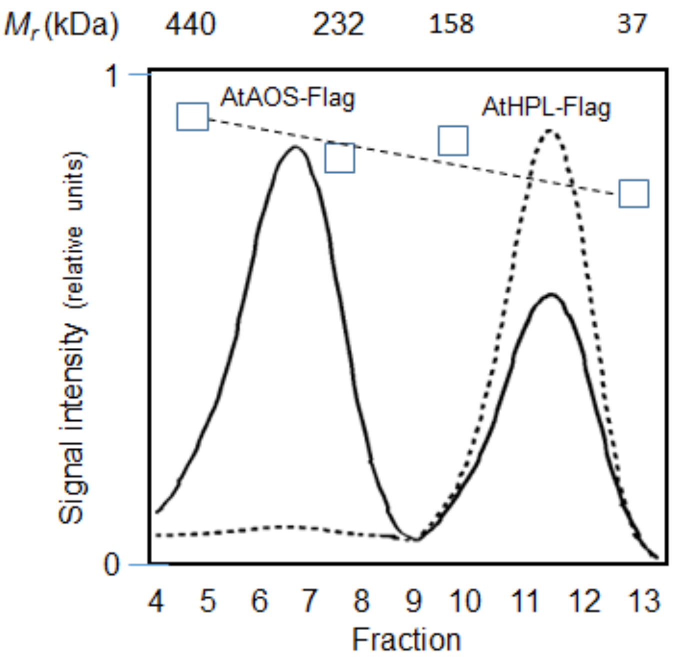

Fig. S3. Size exclusion chromatography of protein complexes formed in plants expressing a Flag-tagged versions of AOS (AOS-Flag, solid line)(A) or HPL (HPL-Flag, dotted line)(B). Positions of apo-ferritin (440 kDa), catalase (232 kDa), aldolase (158 kDa) and carbonic anhydrase (37 kDa) used as molecular size standards are indicated (squares and dotted line). Note that AOS-Flag is present in terms of two complexes of which the higher molecular mass complex eluting in fractions 6 and 7 corresponds to the LOX2-AOS-AOC2 complex (complex I) described in Fig. 4. The lower molecular mass complex eluting in fractions 6 and 7 corresponds to AOS monomers. Vy contrast, HPL-Flag does not form higher molecular mass complex *in planta* and is is present as monomer, eluting in fractions 11 and 12.

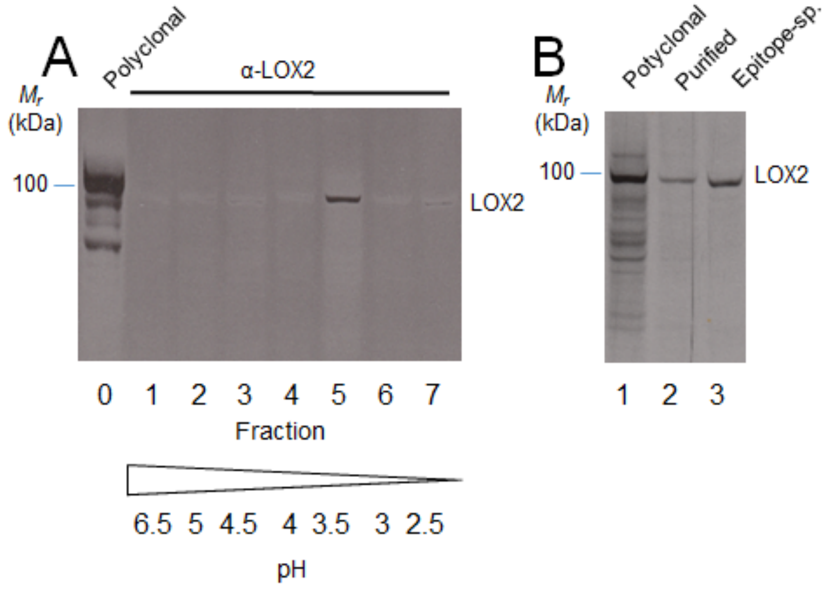

Fig. S4. Identification of monospecific α–LOX2 antibodies. A polyclonal antiserum reacting with multiple LOX protein bands was used to affinity-purify a mono-specific LOX2 antibody. For comparison, an epitope-specific antibody was obtained by immunizing rabbits with a fingerprint peptide of LOX2 distinguishing this from other LOX isoforms. **A**) Elution of LOX2-specific antibodies from the LOX2 affinity column at different pH values, decreasing from 7 to 2.5, as indicated (fractions 1-7). Antibody specificity was tested by immunoprecipitation, using chloroplast protein extract and an aliquot of the eluted fraction. For comparison, the polyclonal LOX antiserum prior to fractionation was used (lane 0). LOX-specific signals were revealed by SDS-PAGE and Western blotting, using the original polyclonal antiserum and an enhanced chemiluminescence (ECL) detection system. The ≈100 kDa ghost band seen in lanes 1-4 as well as lanes 6 and 7 is due to the presence of T7 RNA polymerase carrier protein added to avoid any unspecific degradation of the LOX2 signal during immunoprecipitation. **B**), as **A**), but depicting LOX bands that were precipitated from chloroplast protein extract with the polyclonal antiserum (lane 1), purified mono-specific antiserum (lane 2) and epitope-specific (lane 3) antiserum. Note that the purified and epitope-specific antisera are both specific for LOX2.

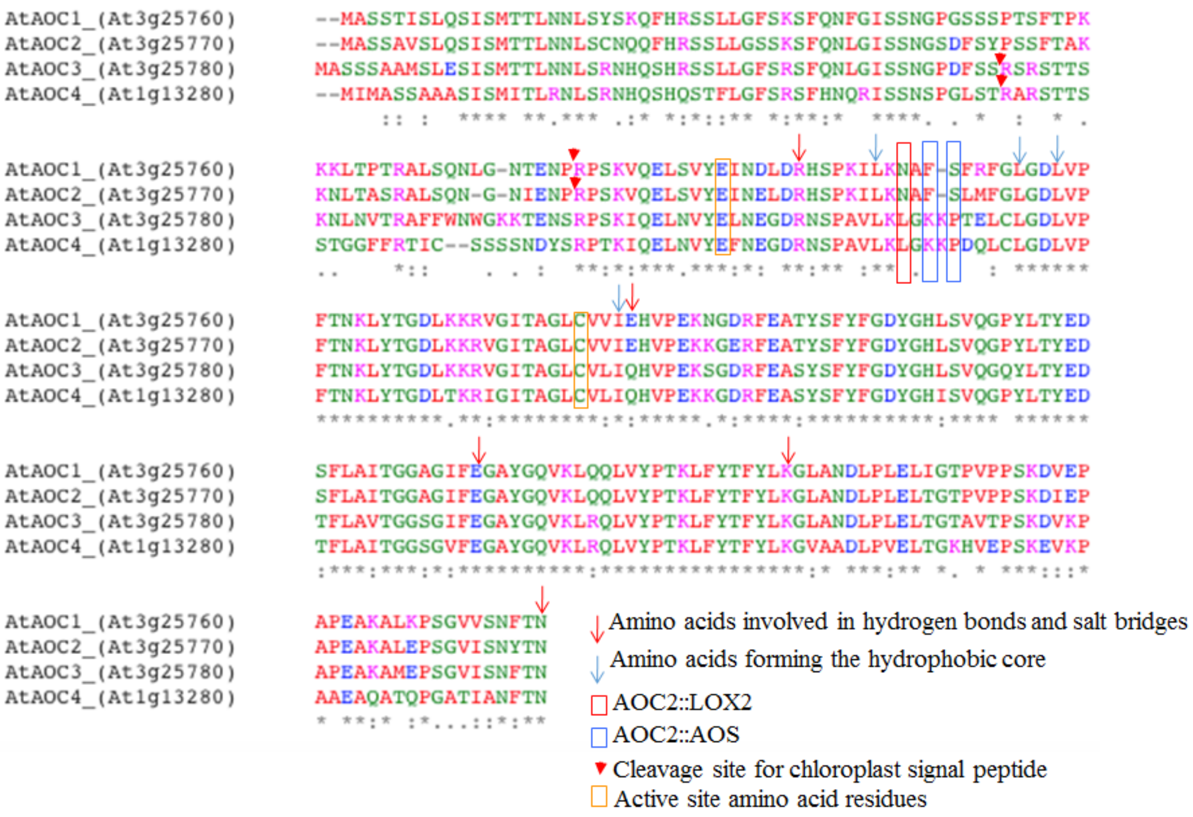

Fig. S5. Multiple sequence alignment of AtAOC1-4. Gene numbers for AOC1-4 are given in parenthesis. Multiple sequence alignment was performed using Clustal Omega (https://www.ebi.ac.uk/Tools/msa/clustalo/). Active site residues were predicted on the basis of Hofmann et al. (2006).

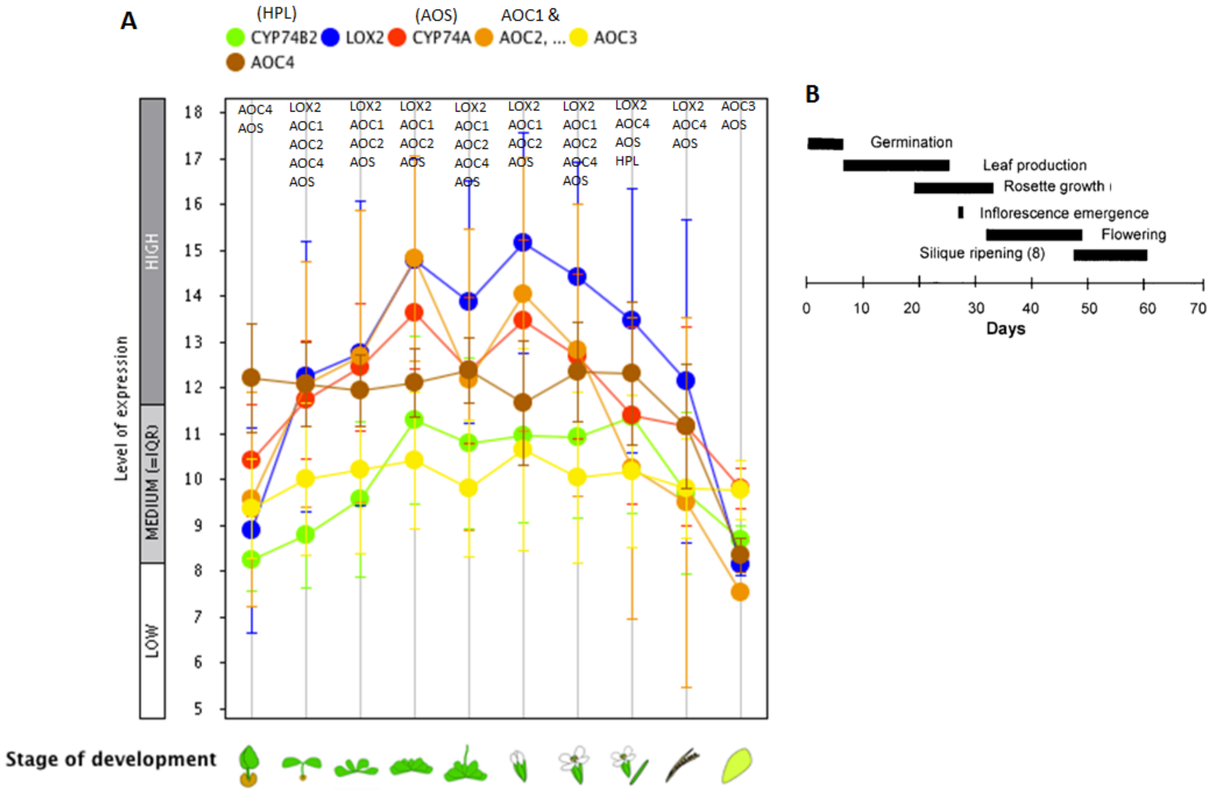

Fig. S6. **A**) Genevestigator* analysis of the expression pattern of the HPL (CYP74B), LOX2, AOS (CYP74A), AOC1/AOC2, AOC3, and AOC4 genes during *Arabidopsis* development. **B**) Schematic display of the chronological progression of major growth stages in *Arabidopsis* (modified from Boyes et al., 2001). *https://genevestigator.com/gv/

Boyes DC, Zayed AM, Ascenzi R, McCaskill AJ, Hoffman NE, Davis KR, Görlach J (2001) Growth stage-based phenotypic analysis of Arabidopsis: a model for high throughput functional genomics in plants. Plant Cell 13:1499-1510.

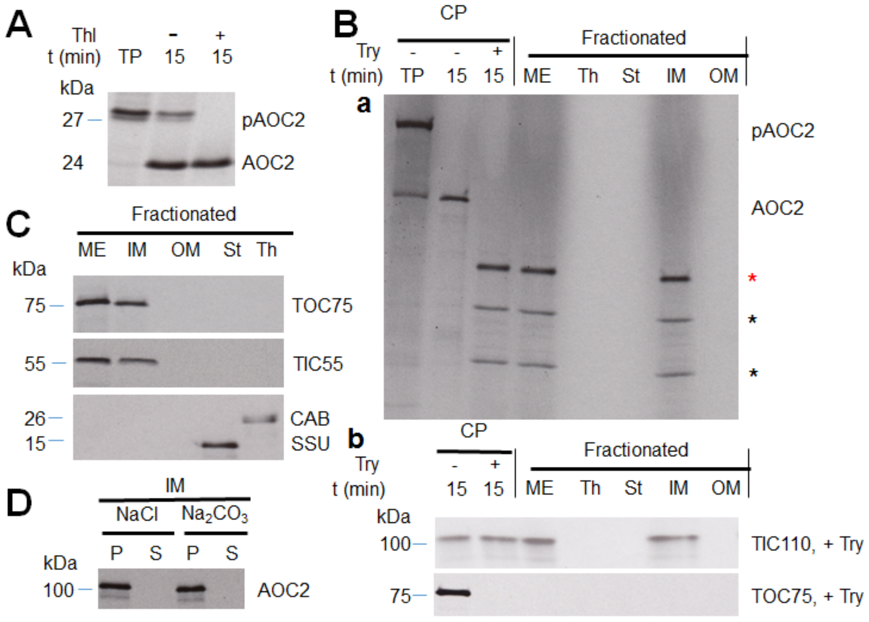

Fig. S7. Import of ^{35}S-*At*AOC2 into isolated *Arabidopsis* chloroplasts and localization of the imported enzyme in the inner envelope membrane. **A**) Import and processing of ^{35}S-*At*AOC2 into isolated Arabidopsis chloroplasts. 'Thl', thermolyin; 'TP', translation product. Positions of precursor protein (pAOC2) and mature protein (AOC2) are indicated. **B**) (panel a), Detection of ^{35}S-*At*AOC2 in trypsin (Try)-treated (+) and non-trypsin-treated (-) chloroplasts (CP) as well as mixed outer and inner plastid envelopes (ME), inner plastid envelope membranes (IM), outer plastid envelope membranes (OM), thylakoids (Th) and stroma (St). 'TP', translation product. Positions of precursor protein (pAOC2) and mature protein (AOC2) are indicated. Starts mark degradation products of ^{35}S-*At*AOC2. Panel b, as panel a, but showing the levels of TIC110 and TOC75 in non-trypsin (Try)-treated chloroplasts (CP) versus trypsin (Try)-treated chloroplasts containing imported ^{35}S-*At*AOC2 and respective subfractions. **C**) TOC75, TIC55, CAB and SSU marker protein distribution across the indicated plastid subfractions. **D**) Salt extractability of imported ^{35}S-*At*AOC2 with 1 M NaCl and 0.1 M Na_2CO_3, pH11, from the inner envelope membranes of chloroplasts. 'P' and 'S' define pellet and supernatant fractions obtained after sedimentation of the membranes.
