# Supplementary material for: Substrate channeling in oxylipin biosynthesis through a protein complex in the plastid envelope of Arabidopsis thaliana
Source: J Exp Bot. 2019 Jan 23;70(5):1483–95. doi: 10.1093/jxb/erz015 (PMC6411374; doi:10.1093/jxb/erz015)
Supplement: Supplementary Figures S8-S12 [file erz015_suppl_supplementary_figures_s8-s12.pdf]

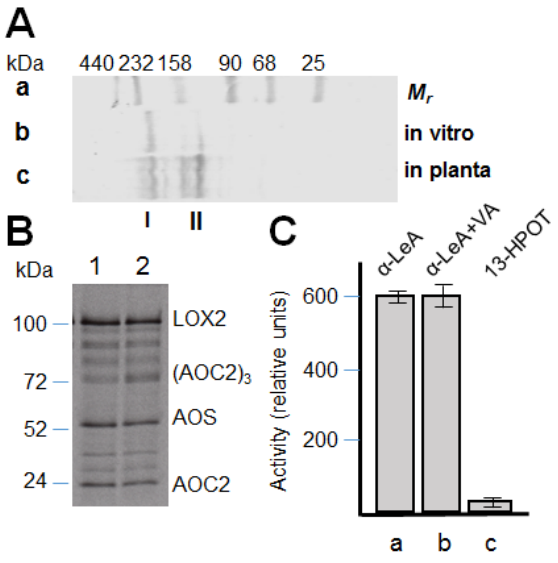

Fig. S8. Reconstitution of *At*LOX2-*At*AOS-*At*AOC2-containing complexes. **A**) Non-denaturing PAGE of reconstituted (b) versus native (c) *At*LOX2-*At*AOS-Flag-*At*AOC2 complexes, as detected by Western blotting using *At*AOS antibody. The two main bands represent complex-bound *At*AOS (band I) and *At*AOS monomers/dimers (band II). Positions of molecular mass markers are indicated (a). **B**) Re-electrophoresis of the proteins contained in band I of the reconstituted (lane 1) and isolated native envelope complexes (lane 2) by SDS-PAGE and identification of proteins was made by Coomassie staining and protein sequencing. **C**) Enzyme activity measurements carried out with the reconstituted complex using α-LeA, α-LeA+ VA and 13-HPOT as substrates.

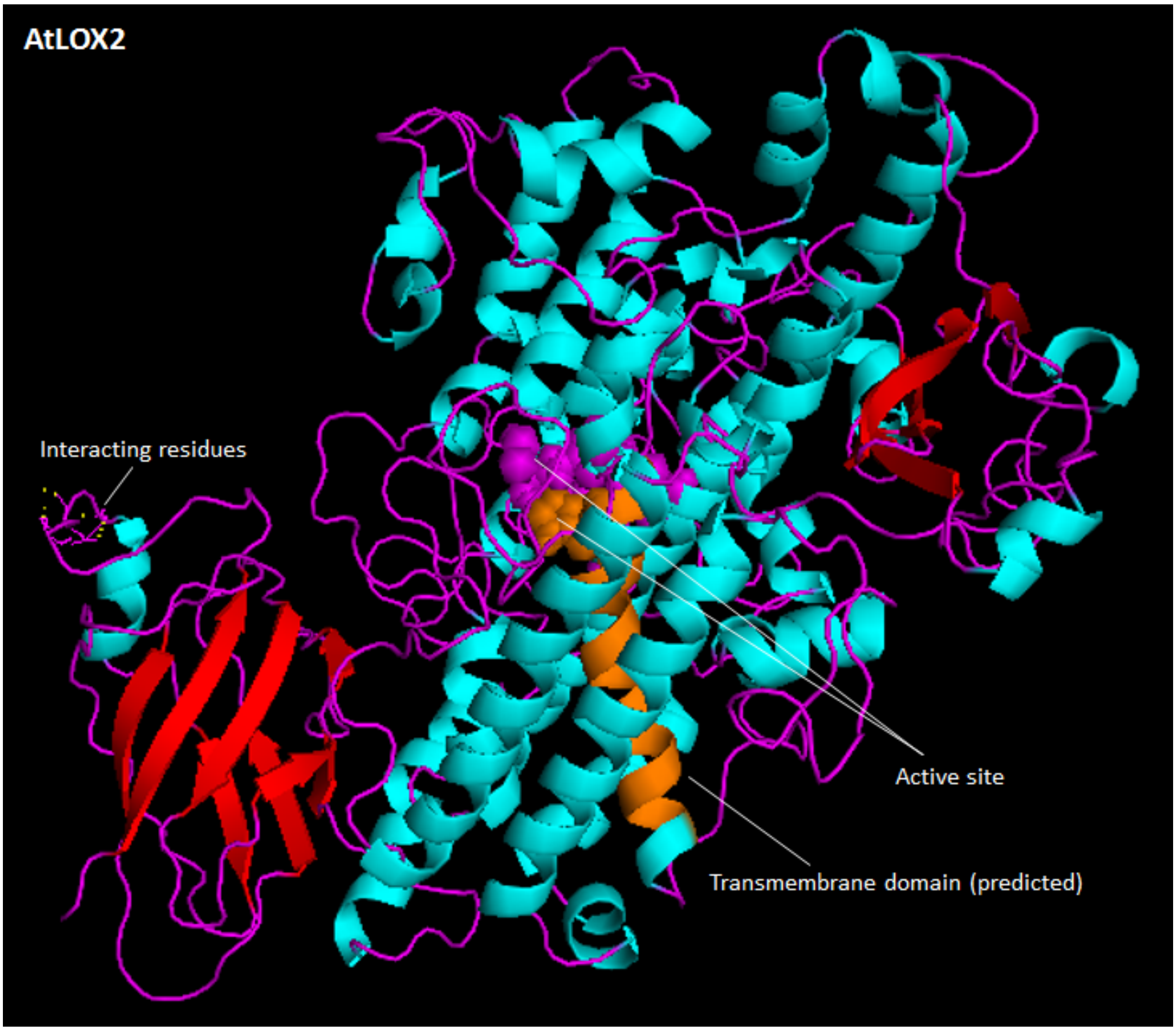

Fig. S9 Ribbon diagram of the *At*LOX2. The structure was modelled using SWISS-MODEL, with soybean lipoxygenase-3 [Protein Data Bank (PDB) ID: 1LNH] as template and visualized using the PyMOL Molecular Graphics System, Version 1.8. The β-strands and α-helices of *At*LOX2 are shown respectively in red and cyan, and connecting loops are shown in magentas. Despite of only 42% sequence similarity with soybean lipoxygenase-3, *At*LOX2 exhibited high structural similarity with it, by having two domains with similar secondary structural elements: a β-barrel N-terminal domain containing highly flexible loops and an α-helix-rich C-terminal catalytic domain. TMpred predicted transmembrane domain was shown in orange. Active site residues are shown by shears, and interacting residues by line. Active site predictions are based on Youn et al. (2006) and transmembrane domain predications are based on TMpred.

Youn B, Sellhorn GE, Mirchel RJ, Gaffney BJ, Grimes HD, Kang C (2006) Crystal structures of vegetative soybean lipoxygenase VLX-B and VLX-D, and comparisons with seed isoforms LOX-1 and LOX-3. Proteins 65:1008-1020

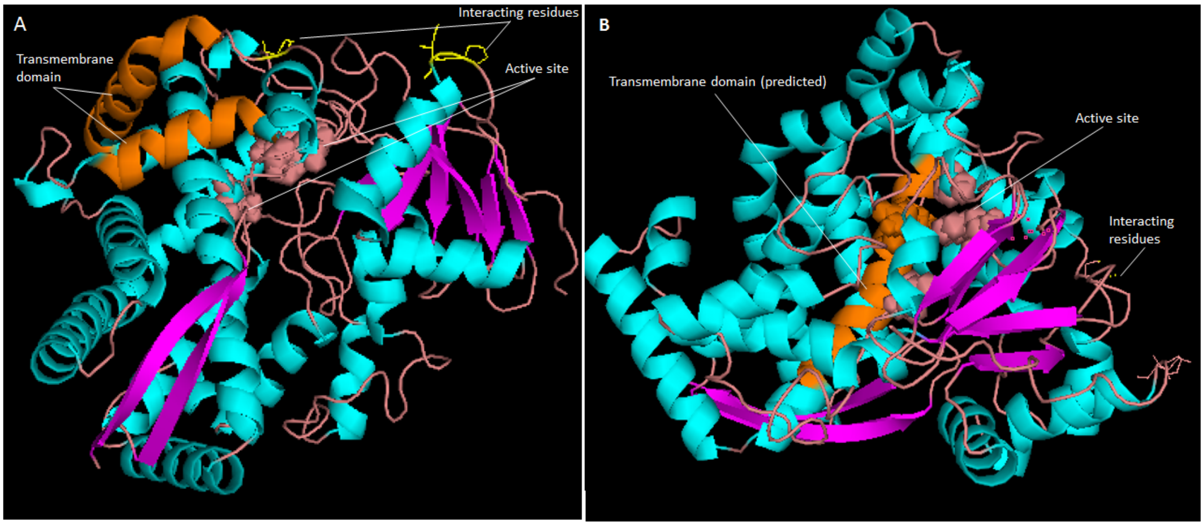

Fig. S10. **A**) The 3D-model of *At*AOS (3CLI) was retrieved from PDB and visualized using the PyMOL Molecular Graphics System, Version 1.8. The β-strands and α-helices in the ribbon diagram of *At*AOS are shown respectively in magentas and cyan, and connecting loops are depicted in salmon. Trans-membrane domains are depoicted in in orange. Active site residues are shown by shears, and interacting residues by line (yellow). Active site residues and trans-membrane domain predications are according to Lee et al. (2008). **B**) as **A**) but highlighting the location of the single TMpred predicted trans-membrane domain and its overlap with active site residues defined by Lee et al. (2008).

Lee DS, Nioche P, Hamberg M, Raman CS (2008) Structural insights into the evolutionary paths of oxylipin biosynthetic enzymes. Nature 455:363-368.

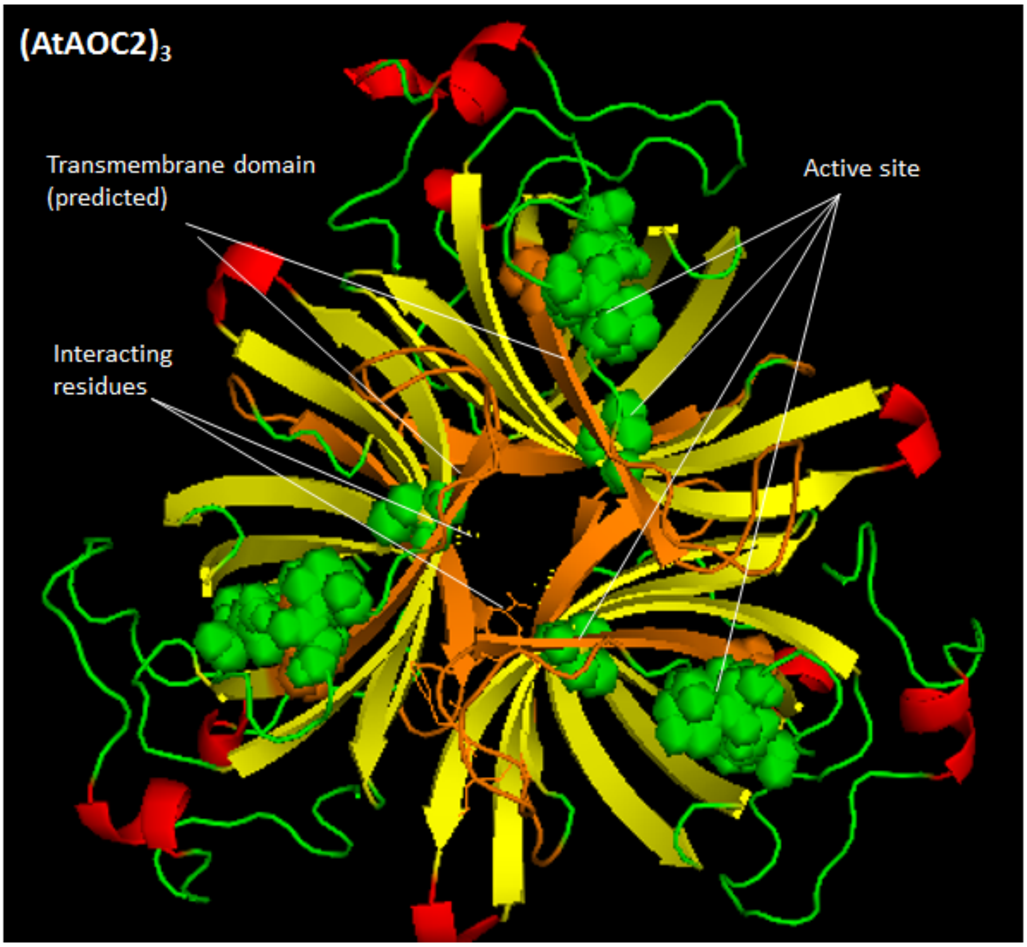

Fig. S11. Ribbon diagram of the *At*AOC2 trimer. The 3D-model of *At*AOC2 (2GIN) was downloaded from PDB and visualized using the PyMOL Molecular Graphics System, Version 1.8. The β-strands and α-helices of *At*AOC2 are shown respectively in yellow and red, and connecting loops are shown in green. TMpred predicted transmembrane domain was shown in orange. Active site residues are shown by shears, and interacting residues by line. Active site predictions are based on Hofmann et al. (2006) and transmembrane domain predications are based on TMpred.

Hofmann E, Zerbe P, Schaller F (2006) The crystal structure of Arabidopsis thaliana allene oxide cyclase: insights into the oxylipin cyclization reaction. Plant Cell 18:3201-3217.

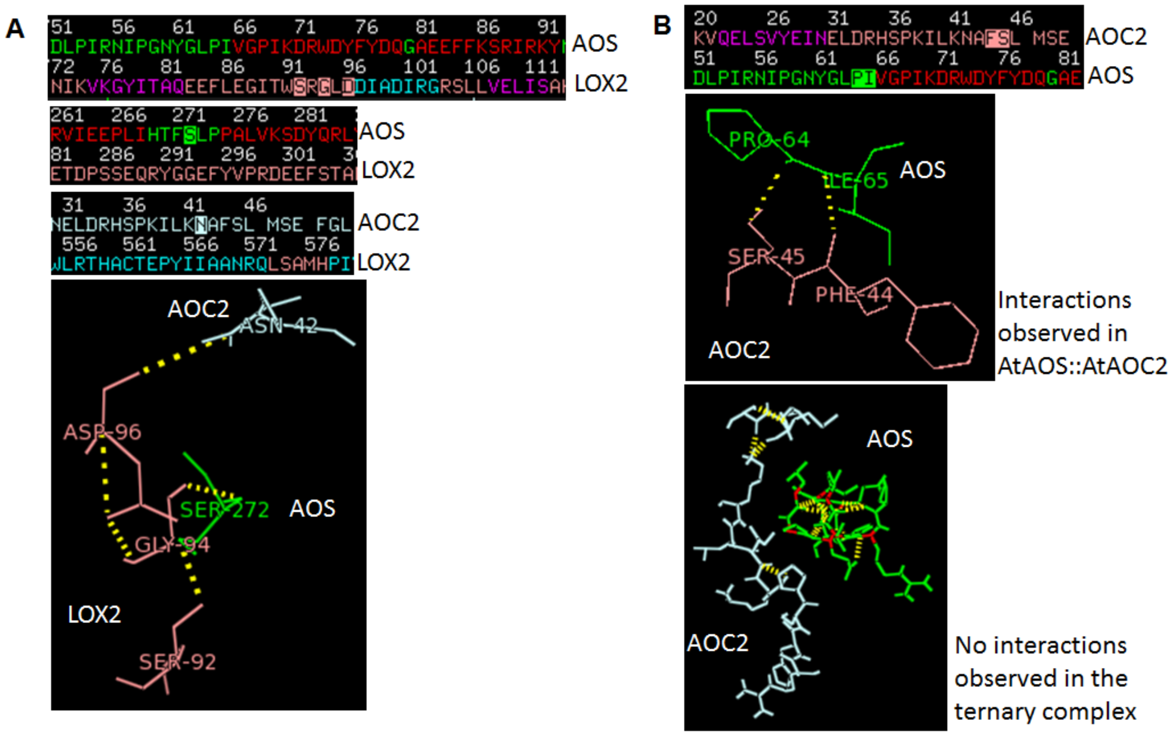

Fig. S12. Identification of amino acid residues involved in the predicted *At*LOX2-*At*AOS-*At*AOC2 interaction model depicted in Fig. 6. Structural modeling describing the *At*LOX2-*At*AOS-*At*AOC2 interaction was carried out using ClusPro. **A**) Specific amino acid residues from *At*AOS, *At*LOX2 and *At*AOC2 that form hydrogen bonds are shown respectively in green, salmon and aquamarine. These interactions involve the following: *At*LOX2:Ser92 & Gly94 - *At*AOS:Ser272, and *At*LOX2:Asp96 - *At*AOC2:Asn42. Amino acid numbering for all proteins is based on the mature protein after removal of the signal peptide. **B**) A part of amino acid sequence and structures of interacting amino acids are shown here to display differential interactions between *At*AOS and *At*AOC2 in the pairwise and complex analyses. Partial amino acid sequences of *At*AOS, *At*LOX2 and *At*AOC2 are shown to display the interacting amino acids (highlighted) and different structural elements (shown in different colors). In the *At*AOS sequence, α-helices are shown in red, β-sheet in yellow, connecting loops in green, and interacting amino acids are highlighted. Similarly, in *At*LOX2 and *At*AOC2 sequences, α-helices are shown respectively in cyan and aquamarine, β-sheets are shown in magenta, connecting loops in deep salmon, and interacting amino acids are highlighted. Amino acid locations in the respective proteins were marked at the top of each sequence.
