# Supplementary material for: Substrate channeling in oxylipin biosynthesis through a protein complex in the plastid envelope of Arabidopsis thaliana
Source: J Exp Bot. 2019 Jan 23;70(5):1483–95. doi: 10.1093/jxb/erz015 (PMC6411374; doi:10.1093/jxb/erz015)
Supplement: Supplementary Dataset S1 [file erz015_suppl_supplementary-dataset-s1.pdf]

SI file III

Densitometric image analysis of the data presented in Figs. 4 and 5.

Original gel blots (Fig. 4) and autoadiograms (Fig. 5) and corresponding contrast-enhanced versions of the images (A1-C1) were scanned using the NIH endorsed ImageJ software, https://imagej.net/)(A2-C2).

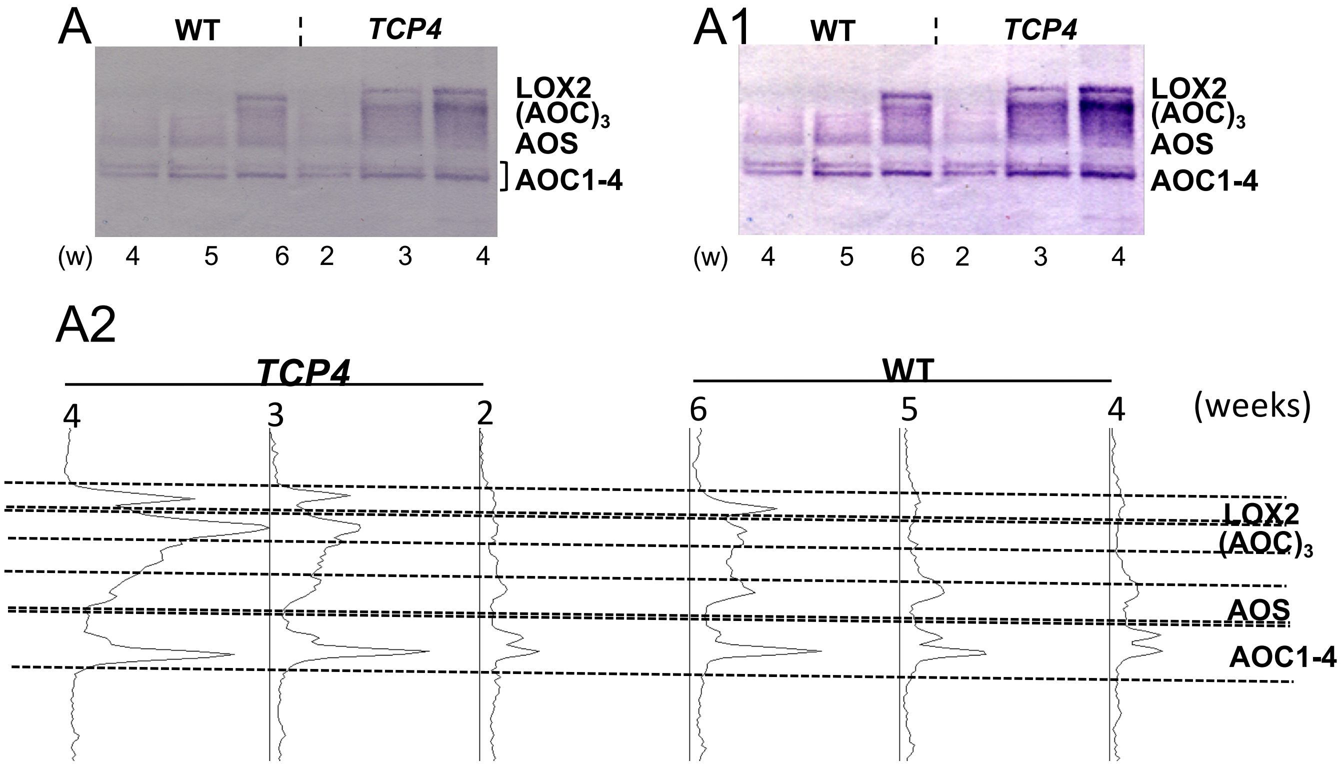

Fig. 4A_scanned

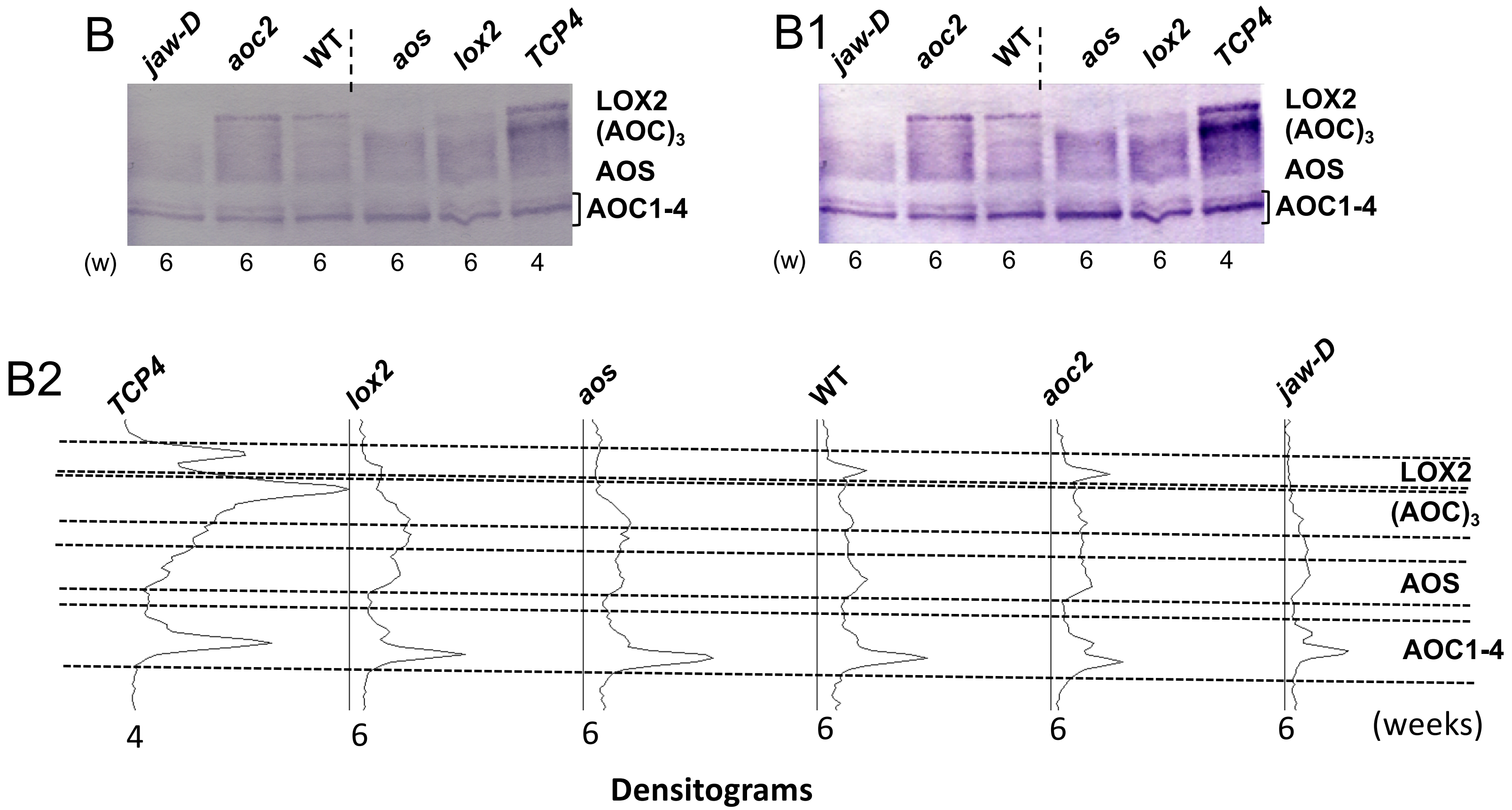

Fig. 4B_scanned

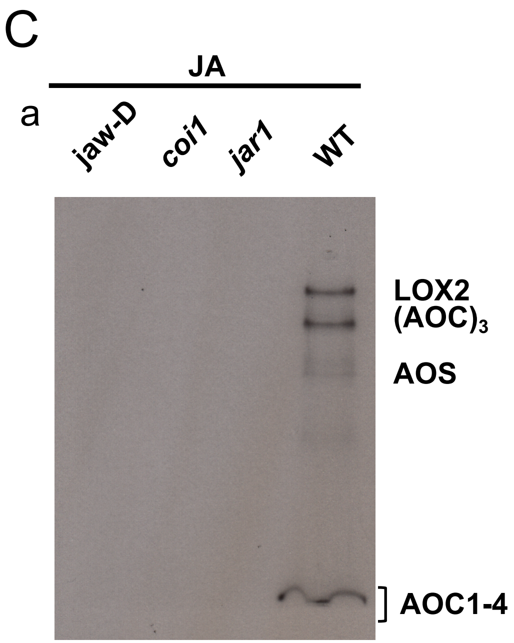

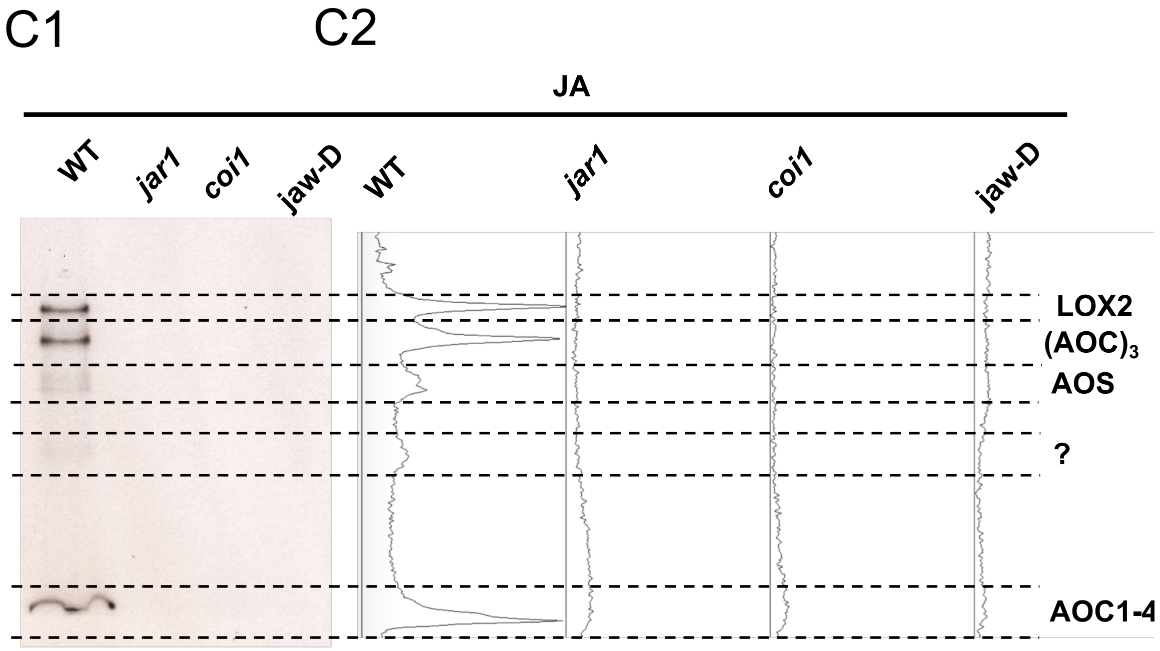

Fig. 4C_scanned

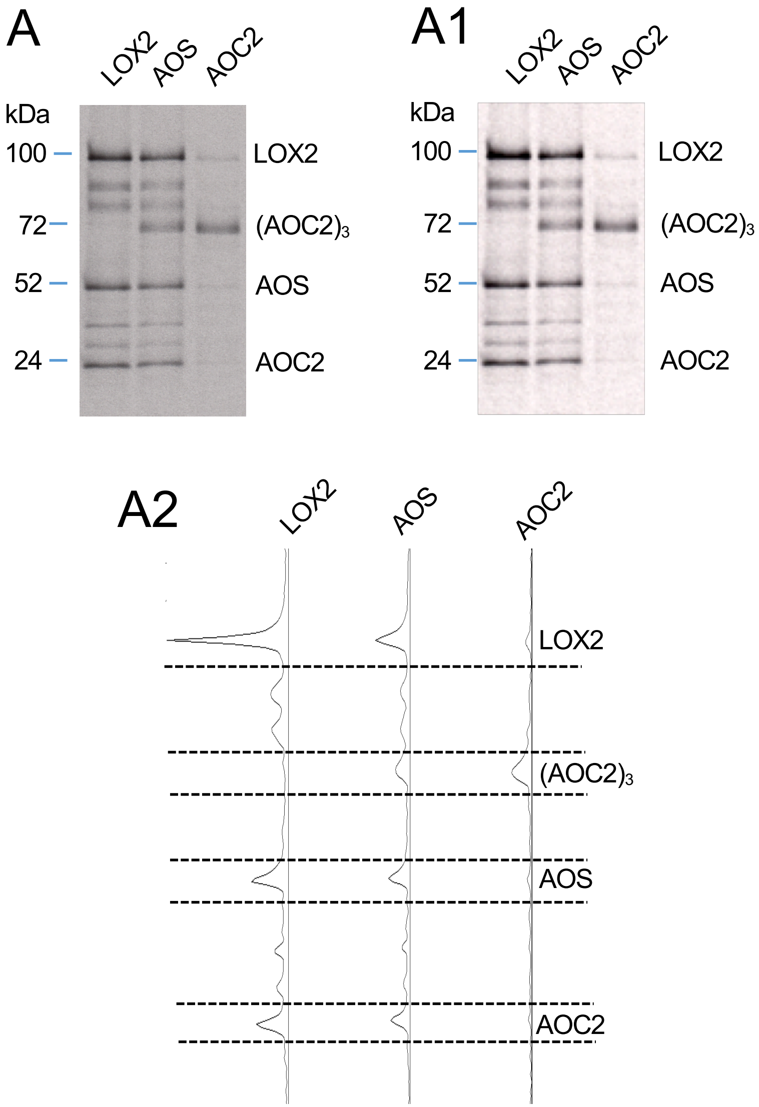

Fig. 5A_scanned
